# Reconstructing avascular necrotic femoral head through a bioactive β-TCP system: From design to application

**DOI:** 10.1016/j.bioactmat.2023.06.008

**Published:** 2023-06-26

**Authors:** Yajie Lu, Xiantao Chen, Xiao Lu, Changning Sun, Minghui Li, Guojing Chen, Zuoyao Long, Yuan Gao, Haoqiang Zhang, Mengquan Huang, Chuanlei Ji, Hongbin Fan, Dong Liu, Yuewen Hao, Hong Wang, Leilei Zhang, Hongmei Zhang, Jianxi Lu, Zhen Wang, Jing Li

**Affiliations:** a Department of Orthopedics, Xijing Hospital, Air Force Medical University, Xi'an, 710032, China; b Department of Clinical Oncology, Xijing Hospital, Air Force Medical University, Xi'an, 710032, China; c The State Key Laboratory of Cancer Biology Biotechnology Center, School of Pharmacy, Air Force Medical University, Xi'an, 710032, China; d Department of Osteonecrosis of the Femoral Head, Luoyang Orthopedic-Traumatological Hospital of Henan Province, Luoyang, 471002, China; e Shanghai Bio-lu Biomaterials Co., Ltd, Shanghai, 201100, China; f Shanghai Technology Innovation Center of Orthopedic Biomaterials, Shanghai, 201100, China; g State Key Laboratory for Manufacturing System Engineering, Xi'an Jiaotong University, Xi'an, 710054, China; h National Medical Products Administration (NMPA) Key Laboratory for Research and Evaluation of Additive Manufacturing Medical Devices, School of Mechanical Engineering, Xi'an Jiaotong University, Xi'an, 710054, China; i Department of Orthopedics, General Hospital of Northern Theater Command, Shenyang, 110000, China; j Department of Orthopedics, The 940th Hospital of Joint Logistics Support Force of People's Liberation Army, Lanzhou, 730000, China; k Department of Medical Imaging, Xi'an Children's Hospital, Xi'an, 710000, China; l Department of Medical Imaging, Xijing Hospital, Air Force Medical University, Xi'an, 710032, China

**Keywords:** Avascular necrosis of the femoral head, β-TCP, Vascularization, Bone regeneration, Clinical trial, Bioadaptability

## Abstract

A variety of techniques have been used for treating avascular necrosis of the femoral head (ANFH), but have frequently failed. In this study, we proposed a β-TCP system for the treatment of ANFH by boosting revascularization and bone regeneration. The angio-conductive properties and concurrent osteogenesis of the highly interconnected porous β-TCP scaffold were revealed and quantified through an *in vivo* model that simulated the ischemic environment of ANFH. Mechanical test and finite element analysis showed that the mechanical loss caused by tissue necrosis and surgery was immediately partially compensated after implantation, and the strength of the operated femoral head was adaptively increased and eventually returned to normal bone, along with continuous material degradation and bone regeneration. For translational application, we further conducted a multi-center open-label clinical trial to assess the efficacy of the β-TCP system in treating ANFH. Two hundred fourteen patients with 246 hips were enrolled for evaluation, and 82.1% of the operated hips survived at a 42.79-month median follow-up. The imaging results, hip function, and pain scores were dramatically improved compared to preoperative levels. ARCO stage Ⅱ disease outperformed stage Ⅲ in terms of clinical effectiveness. Thus, bio-adaptive reconstruction using the β-TCP system is a promising hip-preserving strategy for the treatment of ANFH.

## Highlights

• The porous structure directed neovessels into the encapsulated barren areas, facilitating bone regeneration.
• Mechanical adaptation proceeded during material degradation and tissue regeneration.
• Bio-adaptive reconstruction employing β-TCP system is a promising hip-preserving strategy for ANFH.

## Graphical abstract

**Figure:**
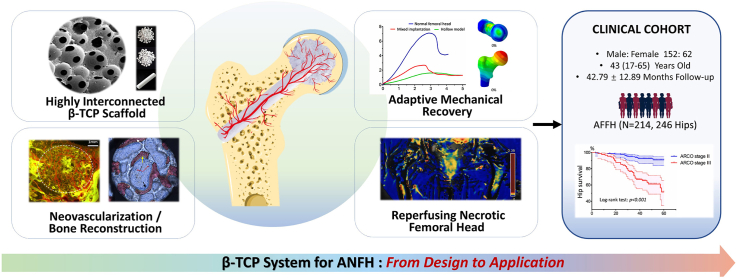

## 1 Introduction

Avascular necrosis of the femoral head (ANFH) is a global health problem with increasing prevalence and mainly affects younger adults between the ages of 20 and 40 years [1,2]. Total hip arthroplasty (THA) is a good therapeutic choice for patients with symptomatic advanced-stage ANFH, but its long-term durability has always been a clinical concern [3]. Hip-preserving therapy is critical for early non-collapsed ANFH in younger patients due to the possibility of a cure. A variety of surgical approaches have been proposed for hip preservation of ANFH, including core decompression [4,5], vascularized or non-vascularized bone-grafting [[6], [7], [8]], osteotomy [9], and stem cell-based therapy [10,11]. However, significant variations in the results obtained from different methods have been seen, and most of them were unsatisfactory. Although the occurrence of ANFH has been attributed to multiple mechanisms, the most critical pathological change is the interruption or reduction of the local blood supply to the femoral head, resulting in tissue ischemia, necrosis, and finally, bone collapse [2]. Thus, necrotic tissue removal and bone grafting seems to be a feasible treatment for ANFH, and previous studies investigated several bone substitutes, including tantalum rods [12], allogeneic bone [13], hydroxyapatite [14], and titanium alloy [15]. However, these substitutes have varying success rates from 31% to 100% [12,[16], [17], [18], [19]], and none of them are widely used in the treatment of ANFH due to their inherent draw backs. For example, porous tantalum rods were used to support subchondral bone, but the histological study showed that the absence of new bone tissue growing into the porous scaffold [20]. The processing and sterilization of allogeneic bone remove cellular components and biological factors, resluted in low bone regeneration potential [21]. Reconstruction of the femoral head, must be precisely bioadaptable [22,23], presenting at least the following three properties. (1)It should show dynamic and active interplay between the implanted materials and the biological microenvironment, which facilitates the reversal of the ischemic state of ANFH and terminates the pathological process of tissue necrosis [24]. (2) It should be mechano-adaptable, generating supports that dynamically match the mechanical properties of the femoral head to be repaired, rather than stress concentration or stress shielding. (3) The materials must be biodegradable and induce tissue regeneration, with a balance between the rate of degradation and that of tissue reconstruction.

β-Tricalcium phosphate (TCP) bioceramic, a synthetic bone graft substitute, is preferably used for bone defects due to its osteoconductive and osteoinductive properties [[25], [26], [27]]. Our previous study showed that the parameters of the porous structure of β-TCP scaffolds, specifically the diameter of the macropores and interconnected holes, significantly influenced vascularization and osteogenesis during bone repair [[28], [29], [30]]. Further, we defined the optimal structural parameters favorable for promoting vascularization and bone regeneration (i.e., macropores with a diameter of 400–500 μm and interconnected holes with a diameter of 100–120 μm) [30]. Our histological study of clinical specimens found that there was rich vascularity and strong biological activity around the necrotic area of the ANFH, representing a reparative change against tissue necrosis. However, this repair was blocked by the sclerotic band. Therefore, the use of porous β-TCP to guide active repair around the necrotic area for femoral head reconstruction to cure ANFH is theoretically feasible and worth trying. We attempted to implement β-TCP scaffolds for the treatment of ANFH in a previous retrospective study and showed preliminary evidence for its effectiveness [31]. However, the theoretical basis of β-TCP treatment for AHFN has not been completely established, and the clinical effects need to be further verified.

In this study, we built a β-TCP system for the bio-adaptive reconstruction of ANFH and assessed it from the perspective of bioadaptability. Porous β-TCP serves as a biodegradable scaffold that interacts with the microenvironment and stimulates bone regeneration [32]. Degradation of the scaffolds and the ingrowth of new bone into the porous structure allows for the restoration of bioadaptable mechanical properties, and eventual return to normal bone tissue. More importantly, β-TCP scaffolds with such optimal porous structures could be utilized to direct reparative neovessels around the grafting areas into the original necrotic region through a vascular conduction effect, thus ending the pathological ischemic state in the necrotic femoral head. This basic and clinical translational research, aimed to (1) establish and refine the theoretical basis for using the β-TCP system to treat ANFH and (2) systematically evaluate the clinical efficacy of the β-TCP system in the hip-preserving treatment of ANFH.

## 2 Methods

### 2.1 Study design

The goal of this study was to create a bioadaptable β-TCP system that would promote revascularization and bone regeneration for hip-preserving treatment of ANFH. The biocompatibility of β-TCP scaffolds was validated in our earlier investigation [[28], [29], [30]]. In this study, we created a β-TCP system consisting of porous β-TCP granules, dense β-TCP granules, and a porous β-TCP rod for the reconstruction of necrotic femoral head. The theoretical basis for treating ANFH using the β-TCP system was established, improved, and validated via *in vivo* experimental and mechanical tests. Then, the clinical efficacy was assessed in a multi-center prospective clinical trial.

#### 2.1.1 Bioceramic system for ANFH: clinical treatment strategy

**Fig. 1:**
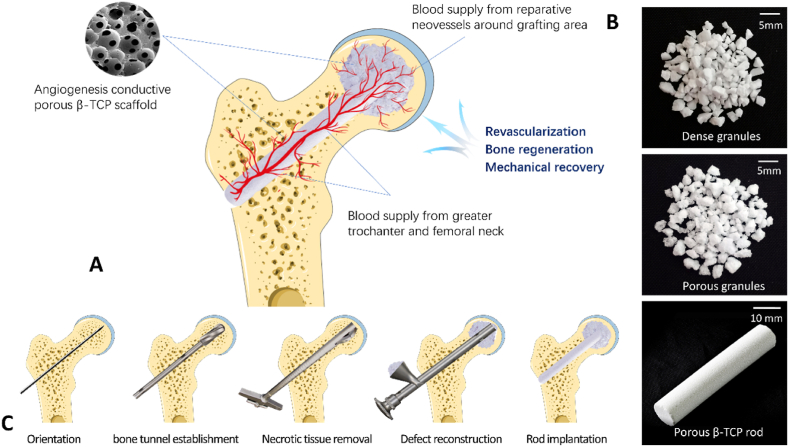
Schematic of the surgical design of the β-TCP system for ANFH treatment. A. Blood vessels from the proximal femur and reparative neovessels around the grafting area were directed into the femoral head through the implanted porous β-TCP scaffold, thus facilitating revascularization, bone regeneration and mechanical recovery; B. The β-TCP system used for ANFH, including dense granules, porous granules, and a porous rod. C. The main surgical procedure, included orientation, bone tunnel establishment, necrotic tissue removal, defect reconstruction, and rod implantation.

We performed single-drilling core decompression and completely scraped the necrotic tissue from the decompression tunnel. A mixture of porous and dense β-TCP granules was compacted into the femoral head, followed by the insertion of a porous β-TCP rod into the decompression channel extending to the bone grafting site of the femoral head (Fig. 1A–C). The porous β-TCP rod acted as a bridge, guiding the blood supply from the greater trochanter and femoral neck into the necrotic regions. Such highly interconnected porous sacffold structures could direct reparative neovessels surrounding the grafting areas towards the implanted β-TCP scaffold inside the femoral head, hence facilitating bone regeneration and adaptive mechanical recovery and, eventually curing ANFH (Fig. 1A). Dense granules, mixed with porous granules, compensated for the mechanical loss of the femoral head after surgery without damaging the structure of the porous scaffold, thus preventing the occurrence of femoral head collapse in the short-term postoperative period (Fig. 1B).

#### 2.1.2 Structure-induced vascularization of the porous β-TCP scaffold *in vivo*

Semi-encapsulated porous β-TCP cylinders were prepared to simulate the ischemic environment of the necrotic femoral head, and then implanted into a rabbit femoral condyle defect model to evaluate its vascularization. We present a unique vascular visualization model to quantitatively assess the porous β-TCP scaffold's characteristics of directing neovessels into barren regions with no nutritional supply, and it is the foundation for employing the β-TCP system to restore the blood supply of the necrotic femoral head. The *in vivo* experiment was conducted at Xijing Hospital according to an approved Institutional Animal Care and Use Committee protocol.

#### 2.1.3 Biomechanical evaluation after implantation

Mechanical support beneath the subchondral bone is the primary element preventing femoral head collapse in ANFH. After β-TCP system implantation, the mechanical characteristics of the proximal femur showed a dynamic and ongoing improvement due to material degradation and new bone formation. In this study, we used compressive tests in pig femur models to assess the mechanical loss due to tissue necrosis and the surgical procedure (necrosis clearance and decompression), as well as biomechanical recovery after β-TCP system implantation. Additionally, three-dimensional (3D) finite-element analysis (FEA) simulations were utilized to evaluate mechanical changes in the proximal femur when the materials were degraded and replaced 0%, 25%, 50%, 75%, and 100% with the new bone.

#### 2.1.4 Multi-center prospective open-label clinical trial

The clinical study was conducted as a multi-center prospective open-label clinical trial, approved by the Institutional Review Board at the participating institution (No.KY20162052-1), and registered at the Chinese Clinical Trial Registry (ChiCTR-ONh-16008989). All patients provided consented and signed informed consent forms. The primary objective of this trial was to assess the clinical efficacy of the β-TCP system in the treatment of ANFH in patients with ARCO stages Ⅱ-Ⅲ by evaluating hip survival, imaging results, pain scores, and hip function.

### 2.2 Material methods: preparation of highly interconnected porous β-TCP scaffold

The slip casting method was used to create the highly interconnected porous β-TCP scaffolds in this study [33]. The primary techniques can be divided into three steps. (1) To establish a porous structure in the following β-TCP scaffold, an organic skeleton composed of polymethylmethacrylate (PMMA) balls (diameter: 400–600 m) was first constructed. Identical PMMA balls were superficially dissolved and joined using an acetone solution in a chemical forming technique. The organic skeleton shrank during this process, and the degree of shrinkage defined the size of the connections between the PMMA balls, which were also the following interconnected holes between the macropores inside the porous β-TCP scaffold. In order to measure the degree of shrinkage of the organic holder, a piston that sliding freely inside the mold was installed in contact with the upper surface of the PMMA beds. An electronic displacement sensor was equipped on the piston to record the settlement of the organic skeleton, which indicated the size of the connections between the PMMA balls. When the theoretical size of the connections reached 110–120 μm, the dissolution reaction was stopped by removing acetone. (2) The β-TCP powders for this study were synthesized by the reaction of a diammonium phosphate solution (NH4)2HPO4 and a calcium nitrate solution Ca(NO3)2·4H2O. β-TCP aqueous slurries made from β-TCP powers were poured into the mold, filling the voids between the PMMA balls, and dried at 60 °C for 12 h (3) In order to achieve the macroporosity within ceramics, the PMMA skeleton was eliminated by annealing samples at 400 °C for 3 h after drying. Then the samples were sintered at 1100 °C for 3 h to reinforce the porous scaffold. Finally, the porous β-TCP scaffolds were cooled to room temperature and sterilized with ethylene oxide.

### 2.3 *In vivo* experiment

Eighteen skeletally mature New Zealand rabbits (2.5 ± 0.5 kg) were included in the *in vivo* experiment. A critical bone defect of 6.00 mm in diameter and 10.00 mm in depth was made in the lateral femoral condyles of each rabbit. To imitate the ischemic environment of ANFH, small cylindrical porous β-TCP scaffolds (diameter, 5 mm; height, 10 mm), semi-encapsulated with a titanium membrane (Zhongbang Titanium Biological Materials Co., Ltd. China), were implanted into the bone defect models. The animals were euthanized for analyses 4, 8, and 12 weeks following implantation.

We developed a novel method for vascular visualization called “fluorescent angiography” to perform an accurate quantitative analysis of blood vessels in implanted scaffold [34]. The method relied on selective vascular perfusion, 3D vessel reconstruction, decalcified hard tissue sections, and fluorescence excitation, enabling the visualization and analysis of the blood vessels in the bone at the capillary level with high resolution. The protocol included the following. (1) Selective vascular perfusion. Firstly, blood vessels of the hind limbs were continuously lavaged with heparin saline (100 U/L) through an “abdominal aorta - hindlimb vascular network - abdominal cardinal vein” perfusion cycle. Next, the hindlimbs were fixed by injecting 300 mL of a 10% neutral formaldehyde solution, and then 50 mL of Microfill MV-117 composite solution (Flow-Tech Inc., Carver, MA) was perfused using an automatic injection pump at a speed of 10 mL/min. Following that, the animals were enthanized and held overnight at 4 °C. The lower femur segments were extracted and fixed with 10% neutral formaldehyde solution for 72 h. The samples were then decalcified with 10% ethylenediaminetetraacetic acid (EDTA)-2Na solution (pH = 7) for 12 days to improve the computed tomography (CT) contrast between bone and perfused vessels. (2) Micro-CT scanning and 3D reconstruction of blood vessels. After decalcification, the specimens were scanned using a micro-CT scanner (Scanco, Switzerland) with a resolution of 1024 × 1024 pixels and a voxel size of 36 μm. The original CT images were imported into Mimics software, and the threshold-based segmentation method was used to reconstruct the 3D views of the blood vessels. (3) Sectioning. Following fixation and dehydration in a graded alcohol series, the samples were embedded in methyl methacrylate (MMA) and sliced into 300 μm thick sections using a microtome (Leica SP 1600; Leica, Solms, Germany). (4) Fluorescence observation. Three slices from the center region of each sample were chosen for observation by fluorescence microscopy. When excited by blue light (430–460 nm), the blood vessels were identified by red color, and the bone tissues were visualized as green.

Van Gieson's (V.G.) staining was conducted to investigate the interplay between angiogenesis and osteogenesis within the implanted scaffolds. We performed double fluorescence labeling by an intraperitoneal injection of tetracycline (20 mg/kg; Sigma) and calcein (10 mg/kg; Sigma) at 8 days and 1 day before enthanization to visualize bone mineralization in the scaffold according to a previously reported protocol [35].

### 2.4 Biomechanical test and 3D FEA

Biomechanical tests were performed on 29 uniform fresh pig femurs using an MTS810 Universal Material Testing Machine (MTS Corp, USA). The mechanical tests consisted of a compression test of the femoral head and a mechanical loading test on simulated surgical samples in the standing posture. In the compression test of the femoral head, 20 femoral head specimens were horizontally truncated 1 cm below the femoral head-neck junction and randomly assigned to create five models (Fig. S1, Supporting Information): normal femoral head; femoral head with a central cavity (diameter, 28 mm); cavity in the femoral head filled with 6 g of porous β-TCP granules (diameter, 1.0–3.5 mm, macropore, 500–600 μm, interconnection, 110–120 μm); cavity fully impacted with 20 g of dense β-TCP granules (diameter, 1.0–3.5 mm, no pores); and cavity impacted with a mixture of β-TCP granules. We previously investigated the mechanical behavior and structural properties of mixed granules with different porous/dense β-TCP proportions (1:10 to 10:1) after compaction in cavity, and we discovered that a mixture of 3 g porous granules and 5 g dense granules can not only avoid the destruction of the main porous structure, but also provide adequate mechanical strength. Therefore, a mixture containing 3 g of porous granules and 5 g of dense granules was used in the biomechanical tests. All samples were subjected to failure testing using axial compression in displacement control at a speed of 1 mm/min. Stress-strain curves were generated online, from which the yield load and stiffness (elastic modulus) were calculated and compared. The mechanical loading test was performed in the standing posture. Nine femoral specimens were truncated from the distal 1/4 and randomly divided into three groups: normal femur; a femoral model that simulated core decompression and lesion curettage (creating a 28-mm-diameter cavity in femoral head) through the tunnel in the femoral neck; and a femur model with mixed β-TCP granules (3 g porous granules plus 5 g dense granules) compacted into the femoral head cavity and a porous β-TCP rod grafted in the decompression tunnel. Four strain gauges were wrapped around the femoral neck to detect micro-deformation at various points. The femoral specimens were anchored at 15° adduction and 10° internal rotation for axial loading to imitate the single-legged stance position (Fig. S2, Supporting Information). Force was then applied vertically to the load-bearing portion of the femoral head at a displacement speed of 1 mm/min until the specimen fractured. Mechanical characteristics were calculated and compared after stress-strain curves and micro-deformation measurements were recorded.

3D FEA was perormed to validate numerical stress-strain behaviors in the biomechanical tests. Images of the femur of a healthy 45-year-old woman were obtained using a 256-slice spiral CT (GE Healthcare, Waukesha, USA) scan. Then, a 3D model of the proximal femur was generated by Mimics software (Version 21.0, Materialise, Belgium). The reconstructed model was imported into Geomagic Wrap 13.0 software (Geomagic Inc., Frankfurt, Germany) for checking, repairing, smoothing, meshing, and fitting the surfaces. Solidwork software (version 2018, Solidwork Corp., Waltham, MA, USA) was used to model the bone graft areas and materials and they were assembled with cortical and cancellous bone models (Figs. S3A–C, Supporting Information). Mechanical analyses were conducted using ABAQUS/CAE software (version 2018, ABAQUS Inc., Providence, RI, USA). Young's modulus and the Poisson ratio of all structures referred in this study were derived from the literature and our tests (Table S2, Supporting Information). A load of 700 N was applied to the weight-bearing area of the femoral head surface, with the distal end of the femur restrained in all directions (Figs. S3D–F, Supporting Information). Von Mises stress distribution and proximal femur displacement distribution were obtained and analyzed further.

### 2.5 Multi-center prospective open-label clinical trial

#### 2.5.1 Participants

A multi-center prospective open-label clinical trial was conducted in five hospitals in China between July 2016 and July 2021. Patients with bilateral or unilateral ANFH were recruited according to the following inclusion criteria: (1) age 16–65 years; (2) imaging diagnosis of ANFH [36]; (3) disease in ARCO stages ⅡA to ⅢC; (4) without previous surgical hip intervention, and (5) volunteered to participate in this trail. The exclusion criteria included: (1) pregnancy; (2) other diseases involving the hip, including developmental dysplasia of the hip (DDH), pelvic or femoral tumor, rheumatoid arthritis, and ankylosing spondylitis; (3) synchronous malignant tumors; (4) surgery intolerance due to severe cardiopulmonary insufficiency; (5) psychiatric disease, and (6) ongoing treatment with high doses of glucocorticoids (>1.0 mg/kg per day).

#### 2.5.2 Surgical protocol

All operations were performed under a standard surgical protocol and used customized surgical instruments (Chinese Patent CN102038544 B) (Fig. S4, Supporting Information). Surgery was performed under spinal or general anesthesia in the supine position. A 2–3 cm incision was made from the tip of the greater trochanter distally. The main surgical protocol consisted of five steps (Fig. 1C). (1) Orientation. A 3-mm Kirschner pin was inserted through the central axis of the femoral neck until it was 0.5 cm beneath the subchondral bone. (2) Bone tunnel establishment. A cannulated hollow reamer was used to broach the tunnel (diameter, 12 mm) along the pin to 0.5 cm below the subchondral bone, and bone marrow cell-rich dust was collected. (3) Necrotic tissue removal. necrosis debridement was performed using a stealth scraper by gradually unfolding the blade and rotating the handle, resulting in the formation of a spherical cavity in the femoral head. (4) Defect reconstruction. Porous β-TCP granules (3 g) and dense β-TCP granules (5 g) (Fig. 1B) were combined and compressed into the cavity using a graft delivery device, and healthy bone marrow collected during tunneling was allowed to mix into the implants to enrich the seed cells for angiogenesis and osteogenesis. (5) Porous β-TCP rod implantation. A porous β-TCP rod (diameter, 10 mm; length, 50–85 mm, Fig. 1B) was introduced into the bone tunnel until it reached at least two-thirds of the femoral head. The incision was cleaned and sutured after fluoroscopic confirmation of implant placement.

#### 2.5.3 Postoperative management

Mild passive hip and knee mobilization began the day following surgery, and physical exercise was encouraged two weeks later. The patients were asked to use double crutches or a wheelchair during the first three months to protect the femoral head from bearing weight. Following this stage, partial weight-bearing with the assistance of crutches was allowed, proceeding to full weight-bearing according to the results of plain radiographs.

#### 2.5.4 Outcome assessment

All patients were evaluated clinically and radiographically prior to treatment, at 1, 3, and 6 months postoperatively, and at 6-month intervals thereafter. The follow-up lasted at least two years or until THA. The visual analogue scale (VAS) was adopted to evaluate pain symptoms before and after treatment. The harris hip score (HHS) was used to assess hip functional outcomes, and graded into four grades: excellent (HSS >90), good (80 < HSS ≤90), moderate (70 < HSS ≤80), and poor (HSS ≤70).

Plain radiography was used to assess radiographic progression, which was defined as progressive collapse, necrotic enlargement, joint space narrowing, or increases in the ARCO stage. Dynamic contrast-enhanced magnetic resonance imaging (DCE-MRI) or SPECT/CT scans were performed on patients who agreed to be investigated to assess the vascularity status of the femoral head before and after surgery. Clinical failure was defined as symptom wrosening, resulting in a strong desire for THA or conversion to THA.

### 2.6 Statistical analysis

Statistical analyses were conducted using GraphPad Prism software (version5.0, GraphPad Software Inc., USA) and IBM SPSS software (version 17.0, IBM SPSS inc., USA). The enumeration data (e.g., hip survival rate and complications) are presented as percentages, and the measurement data (e.g., yield load, stiffness, vascularization indexes, osteogenesis indexes, HHS, K^trans^, and iAUC) are presented as th mean (SD) or median (range). The Student's t-test (unpaired and two-tailed), chi-squared test, or one- or two-way analysis of variance (ANOVA) was used for group comparisons. Hip survival was analyzed using the Kaplan-Meier curve and log-rank test. Values were deemed significantly different at p < 0.05 for all analyses.

## 3 Results

### 3.1 Highly interconnected structure of the porous β-TCP scaffold

**Fig. 2:**
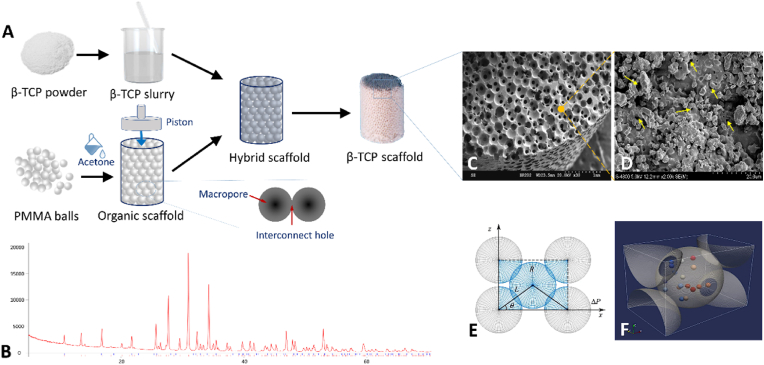
Preparation of porous β-TCP scaffold and its main characteristics. A. The method of spatially controlled molding by the slip-casting process for porous β-TCP scaffold preparation; B. X-ray diffraction (XRD) patterns of the β-TCP scaffold. C. A three-dimensional view of the highly interconnected structure in the porous β-TCP scaffold taken by scanning electron microscopy (SEM); D. SEM image showing the micropores (with the size of 200 nm–1000 nm) of the matrices of porous β-TCP scaffold. The yellow arrows indicate the location of micropores. E-F. Schematic diagram of the “basic repair unit”. (E) The “basic repair unit” generated by theoretical calculations. Each pore contacted the adjacent four surrounding pores in a single plane. Such an interconnected structure could be parameterized by the ball center distance (L), the radius of balls (R), and the included angle (θ); (F) Stereo view of the repair unit, cells (marked as colored pellets) moved from one pore to another through the interconnected holes and deposited on the concave pore walls, forming the bed of seeds for tissue regeneration.

Several types of β-TCP scaffolds were prepared for use in the experimental and clinical studies, including porous β-TCP rods (cylinder diameter: 10 mm, length, 50–85 mm), small porous β-TCP rod (cylinder diameter, 5 mm; length, 10 mm, for *in vivo* experiment), porous granules (irregular, 1–3.5 mm), and dense granules (irregular, 1–3.5 mm) (Fig. 1, Fig. 2A). The X-ray diffraction (XRD) pattern of the sintered porous samples showed that the scaffolds were exceedingly pure, with no impurity phases, and fully matched the standard β-TCP card (JCPDS 09–0169). (Fig. 2B). The microstructure of the porous β-TCP scaffolds was confirmed to have outstanding homogeneity by electron microscopy analysis, with a porosity of 75% ± 10%, macropores of 512.4 ± 121.4 μm in diameter, and interconnected holes of 116.4 ± 14.6 μm (Fig. 2C). The rate of connectivity between the pores was 100%. Dense scaffolds and porous scaffold matrices presented the same geometrical features as micropores with sizes of 200 nm–1000 nm (Fig. 2D). Every pore in each plane was connected to the adjacent four pores, forming the “basic repair unit” of the connected structure (Fig. 2E and F), which was defined as the minimum functional unit during the process of tissue repair. Computer simulation showed that cells moved between the pores through interconnected holes and preferred to be deposited on the concave walls of the pore where the progenitor cells of the bone and blood vessels gathered, forming a bed for endogenous tissue regeneration (Fig. 2F). This highly interconnected structure of the porous β-TCP scaffold also facilitated the circulation of body fluids and promoted material degradation.

### 3.2 Adaptive tissue regeneration - neovascularization guided by porous structures

**Fig. 3:**
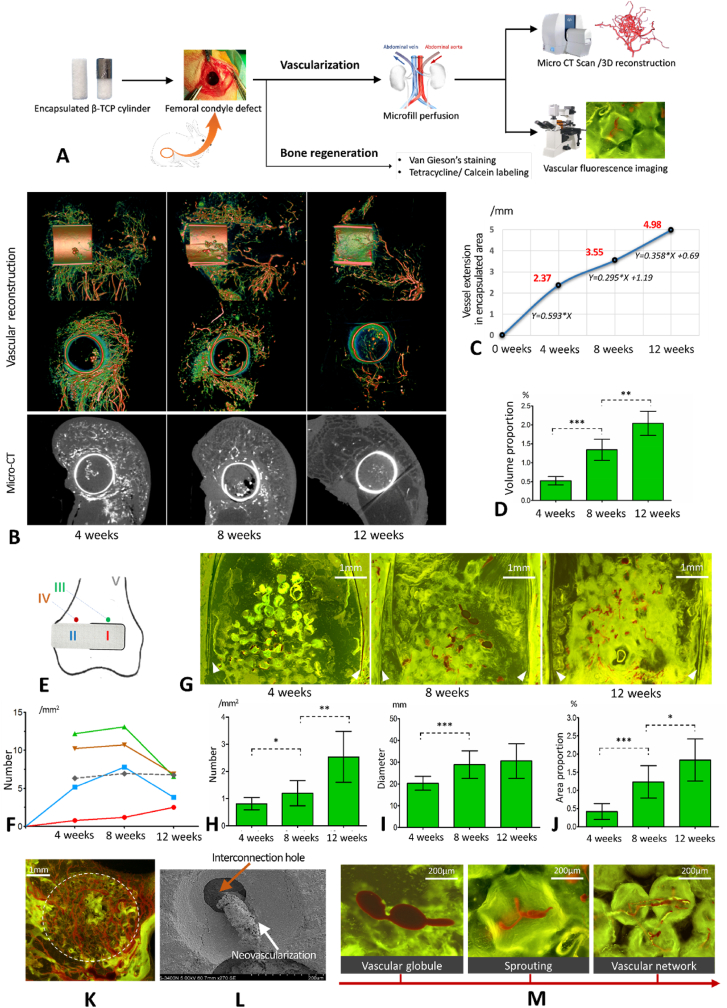
Results of the *in vivo* neovascularization experiment. A. Flow chart of the *in vivo* animal experiment. B. Micro-CT scan and 3D reconstructions of blood vessels. C. The depth of the vessels extended into the encapsulated region. D. Volume proportion of the newly formed blood vessels in the encapsulated region 4, 8, and 12 weeks after implantation. E. Area division for the quantitative analysis of vascularization. Area Ⅰ: encapsulated β-TCP rod; area Ⅱ: unencapsulated β-TCP rod; area Ⅲ: the area around the encapsulated region; area Ⅳ: the area around the unencapsulated region; and area Ⅴ: normal cancellous bone. F. Number of blood vessels in areas Ⅰ to Ⅴ. The number of blood vessels in the encapsulated area was significantly lower than that of the non-encapsulated area at 4 weeks and 8 weeks; G. Fluorescence angiography of the encapsulated area. The white triangles indicate the positions of the titanium membrane. H. Number of blood vessels in the encapsulated area at 4, 8, and 12 weeks after implantation. I. Diameter of blood vessels in the encapsulated region at 4, 8, and 12 weeks after implantation. J. Vessel area proportion in the encapsulated region 4, 8, and 12 weeks after implantation. K. Angiography under fluorescent background in the unencapsulated area shows abundant vascularization at 4 weeks; L. Electron microscopy shows that newly formed blood vessels travel through the interconnected holes. M. The angiogenic process of encapsulated and unencapsulated areas in porous β-TCP scaffold. *p < 0.05, **p < 0.01, ***p < 0.001.

The *in vivo* experimental procedure is shown in Fig. 3A. Blood vessels in and around the semi-encapsulated porous β-TCP scaffold were clearly marked in micro-CT 3D reconstructions (Fig. 3B, movie S1 Supporting Information). In the 4th week, there were abundant newly formed blood vessels in the unencapsulated region of the implanted rod, and those vessels started extending from the unencapsulated region to the encapsulated region (Fig. 3B). In the 8th week, vascular clusters were visible in the encapsulated region, and by the 12th week, new blood vessels had penetrated the entire encapsulated region (Fig. 3B and C). The vascular volume proportion (vascular volume/the whole volume of encapsulated rod) measured in the 3D models was 0.52%, 1.34%, and 2.04% in the 4th, 8th, and 12th weeks, respectively, with a significant difference (p < 0.001) (Fig. 3D). To further quantify the vascularization of the β-TCP rod, the depth of the vessel extensions, the number of vessels, the diameter of the vessels, and the vessel area proportion were measured both in two-dimensional micro-CT images and angiography images under fluorescence, based on the implant-centered area divisions shown in Fig. 3E. A large number of reparative vessels appeared in the host bone around the implant in the 4th week, providing a favorable microenvironment for the vascularization of the implanted β-TCP rod. Following that, these vessels gradually receded as vascularization was completed in the 8th to 12th weeks (Fig. 3B and F). According to the quantitative analysis, the first four weeks had the fastest vascular extension into the encapsulated area, with an ingrowth rate of 0.593 mm per week (Fig. 3C). The rate of vascular ingrowth slowed slightly after four weeks, but it was characterized by gradual vascular maturation and remodeling (Fig. 3B, C, and 3G). Significant increases in the number, diameter, and vessel area proportion in the encapsulated area (area Ⅰ) were observed from the 4th week to the 12th week (Fig. 3H–J, p < 0.05), confirming the porous β-TCP scaffold's reliability in guiding blood vessels to grow into areas with poor blood supply. The unencapsulated β -TCP region (area Ⅱ) received sufficient blood supply within four weeks, and the number of blood vessels reached saturation in the 8th week, followed by a gradual decline from the 8th to the 12th week (p < 0.05) (Fig. 3F), but with an increase in vessel diameter, indicating a transition from dysfunctional reparative vessels to functional mature vessels.

The angiogenic process in the porous β-TCP scaffolds was first identified using fluorescence angiography. Angiogenesis in the unencapsulated areas originated from the surrounding normal tissue and aggregated into a dense network (Fig. 3K). Encapsulated areas exhibited angiogenesis similar to unencapsulated areas but with a delay (Fig. 3B and F). During the early stage of angiogenesis, only a few tissues occupied the pores, forming vascular globules that then sprouted and travelled through the interconnected holes between the pores to build a vascular network (Fig. 3L and M). Accompanied by material degradation and tissue entry, these immature vascular networks were gradually remodeled into mature and functional vessels.

### 3.3 Adaptive tissue regeneration - bone reconstruction occurs concurrently with neovascularization

**Fig. 4:**
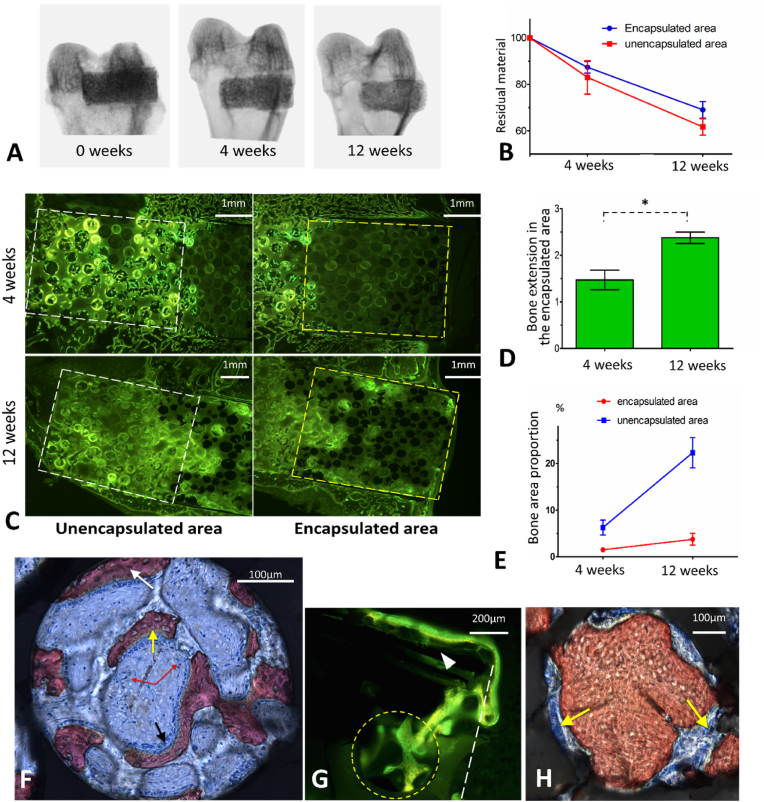
Results of *in vivo* for bone regeneration concurrent with neovascularization. A. X-ray transmission of the specimens shows the degradation of the implanted porous β-TCP scaffold. B. Degradation rate of the β-TCP scaffold in encapsulated or unencapsulated region. C. Tetracycline/calcein labeling for bone regeneration analysis in encapsulated and unencapsulated areas. D. The depth of new bone extended into the encapsulated area 4 weeks and 12 weeks after implantation; E. Bone area proportion of encapsulated or unencapsulated areas at 4th and 12th week. F. Osteogenic patterns in one pore (V.G. staining). The white arrow indicates osteogenesis extending from other pores, the yellow arrow indicates *in situ* osteogenesis and the red arrows indicate blood vessels. G. Tetracycline/calcein labeling shows that the host bone outside the encapsulated area extends across the titanium membrane into the encapsulated area. The white triangle indicates the titanium membrane and the yellow circle indicates one macropore. H. Remodeling process of the newly formed bone (V.G. staining). The yellow arrows indicate osteoclasts. *p < 0.05, **p < 0.01, ***p < 0.001.

The bone repair process was initiated immediately after implantation, but it lagged slightly behind neovascularization. X-ray transmission analysis showed that both the encapsulated and unencapsulated areas demonstrated progressive material degradation, indicating active communication between the β-TCP implants and the microenvironment (Fig. 4A). However, the degradation rate in the encapsulated area was slower than that in the unencapsulated area (p < 0.05) due to the lack of lateral communication (Fig. 4B). We confirmed new bone formation in the encapsulated area by tetracycline/calcein labeling (Fig. 4C). The newly formed bone extended to a depth of 1.471 ± 0.148 mm and 2.376 ± 0.088 mm into the encapsulated area at the 4th and 12th weeks, respectively (Fig. 4D). The bone area proportion was 1.51% in the 4th week and increased to 3.75% in the 12th week (p < 0.05) (Fig. 4E).

The bone regeneration process was identified in the porous β-TCP scaffold. Two distant osteogenic patterns coexisted: 1) periosteum extension, in which the periosteum of the host bone extended into the superficial pores, adhered to the pore wall to form new bone, and passed through the interconnected holes to join osteogenesis (Fig. 4F and G); and 2) *in situ* osteogenesis in which bone marrow components rich in mesenchymal stem cells (MSCs) and vascular progenitor cells filled the pores after implantation. MSCs differentiated into osteoprogenitor cells, followed by osteoblasts to promote osteogenesis, and vascular progenitor cells differentiated into endothelial cells, resulting in the formation of blood vessels (Fig. 4F). Numerous calcium and phosphorus elements were released during material degradation, which induced the recruitment of osteogenic factors and promoted new bone mineralization (Fig. S5, Supporting Information). In the later stage, as the material degraded, many osteoclasts participated in bone remodeling, thus forming stable woven bone tissue (Fig. 4H). In summary, tissue regeneration in the porous β-TCP scaffold could be characterized by three stages, cell interconnection, blood vessel interconnection, and bone interconnection (Fig. S5, Supporting Information).

### 3.4 Mechanical adaptation of the femoral head following β-TCP implantation

**Fig. 5:**
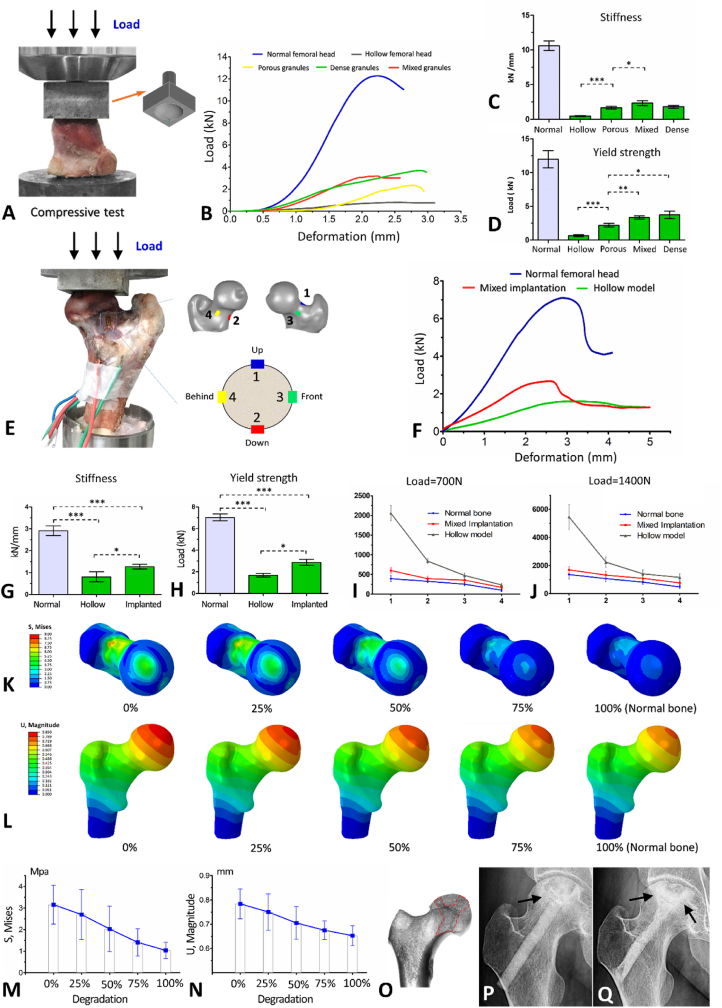
Mechanical analysis results. A. Compressive test model. B. Stress-strain curves of femoral head compressive tests. C. Stiffness of the normal, hollow, porous, mixed, and dense granules implanted in the femoral head model. D. Yield load of the normal, hollow, porous, mixed, and dense granules granules implanted in the femoral head model. E. Mechanical tests of surgical simulation samples in the standing posture. The number of “1–4” represented four strain gauges adhered around the femoral neck. F. Mechanical stress-strain curves of surgical simulation models in the standing position. G. Stiffness in the mechanical tests of normal, hollow, and bioceramic implanted proximal femur models in the standing position. H. Yield load of the normal, hollow, and bioceramic implanted proximal femur models in the standing position for mechanical tests. I. Micro-deformation of the four strain gauges under a load of 700 N. J. Micro-deformation of the four strain gauges under a load of 1400 N. K. 3D FEA. Von Mises stress cloud diagram under different degradations of the implanted β-TCP scaffold. L. 3D FEA. Displacement cloud diagram under different degradations of the implanted β-TCP scaffold. M. Changes in von Mises stress of the femoral head under different degrees of degradation (0, 25%, 50%, 75%, and 100%). N. Changes of displacement of the femoral head under different degrees of degradation (0, 25%, 50%, 75%, and 100%). O–Q: Clinical validation of mechanical recovery. (O) The bone trabeculae of the normal femoral head were regularly and tightly arranged in the direction of stress. The red frame indicates the arrangement of the bone trabeculae subjected to compressive stress in the femoral head. (P) The trabecular structure was destroyed by necrotic lesions (indicated by the arrow). (Q) Five years after β-TCP treatment, the regenerated bone trabeculae (indicated by arrows) were rearranged in the direction of normal stress. *p < 0.05, **p < 0.01, ***p < 0.001.

A substantial reduction in mechanical strength was observed after bone curettage in the femoral head, and only 4.36% stiffness and 5.34% yield strength remained (Fig. 5A–D). Compactly implanting the β-TCP scaffold into the femoral head cavity could partially compensate for the mechanical loss caused by curettage. Specifically, the yield strength of the femoral head impacted with porous, mixed, and dense β-TCP granules was restored to 18.35%, 27.80%, and 31.12% of normal bone, respectively, and stiffness was restored to 15.65%, 22.02%, and 16.91% (Fig. 5C and D). We collected the β-TCP granules after the compression test to evaluate whether the porous structure of each group was significantly damaged during grafting and compressing. As illustrated in Fig. S6, Supporting Information, practically all of the granules in the porous group were fractured, but the mechanical structure of the porous granules in the mixed group was mostly intact. The findings indicated that the mixture of porous and dense β-TCP granules was the best choice for filling the femoral head cavity after necrotic bone curettage to balance mechanical compensation and retain the interconnected porous structure for angiogenesis.

The mechanical test results of surgical simulation samples revealed that the β-TCP system for ANFH restored 40.89% of the yield strength and 44.13% of the stiffness of the proximal femur in the initial postoperative stage (Fig. 4E–H). We also discovered that the mechanical yield of the proximal femur was usually accompanied by femoral neck fractures. During vertical mechanical loading, the top region of the femoral neck was subjected to high tensile stress, which was the weakest proximal femoral mechanics position. The decompression tunnel also caused a reduction in the mechanics of the femoral neck, leading to a risk of femoral neck fracture under excessive load. However, after the implantation of the β-TCP system, the mechanical strength of the femoral neck was compensated to a certain extent, reducing this risk of fracture (Fig. 5F,I, and 5J). There was no significant difference (p > 0.05) in micro-deformation between the operation and normal bone group under the pressure of body weight (700 N) or double body weight (1400 N), indicating the initial safety of this system for patients with ANFH (Fig. 5I and J).

The results of 3D FEA are shown in Fig. 5K-N. The von Mises stress cloud diagram showed that the stress distribution was relatively homogeneous at the normal femoral head (1.037 ± 0.375 Mpa) without an apparent stress concentration region (Fig. 5K). After spherical clearance of the femoral head and the creation of the bone tunnel, the von Mises stress of the femoral head increased substantially to 4.662 ± 0.949 Mpa (p < 0.001). Unfortunately, the load-bearing area of the femoral head formed an obvious stress concentration region, with a maximum value of 6.165 Mpa. The stress values of the femoral head were lowered after implanting the β-TCP system (3.155 ± 0.899 Mpa, p = 0.003). With the continous material degradation and bone regeneration, the von Mises stress of the implanted femoral head dynamically reduced, eventually approximating that of the normal femoral head (100% degradation) (Fig. 5K and M), and the stress concentration region also shrank gradually. Similar to the mechanical test results, the 12 o'clock and 6 o'clock regions of the femoral neck were mechanically concentrated on the von Mises stress cloud diagram. However, such concentration was alleviated as the materials degraded and new bone formed (Fig. 5K). The displacement cloud diagram showed that maximum displacement occurred in the weight-bearing area of the femoral head (Fig. 5L). In the cavity model, the average displacement of the weight-bearing area of the femoral head was 0.890 ± 0.080 mm, which was significantly greater than that of the normal femoral head (0.653 ± 0.041 mm, p < 0.001). β-TCP material implantation could partially compensated for such displacement (0.784 ± 0.062 mm, p = 0.023). Similarly, as material degradation and bone regeneration occurred, femoral head deformation decreased, eventually returning to normal bone values (Fig. 5L and N).

Adaptive mechanical recovery was also validated clinically in patients with ANFH who have underwent β-TCP implantation. We observed adaptive changes in the bone microstructure. In the process of material degradation and new bone formation, the regenerated bone trabeculae were arranged and remodeled in the direction of the load stress to reconstruct the mechanical microstructure destroyed by necrotic lesions, which supported the femoral head and avioded the collapse of the entire structure (Fig. 5O-Q).

### 3.5 Clinical translation – outcomes of the clinical trial

#### 3.5.1 Characteristics of the enrolled patients

**Fig. 6:**
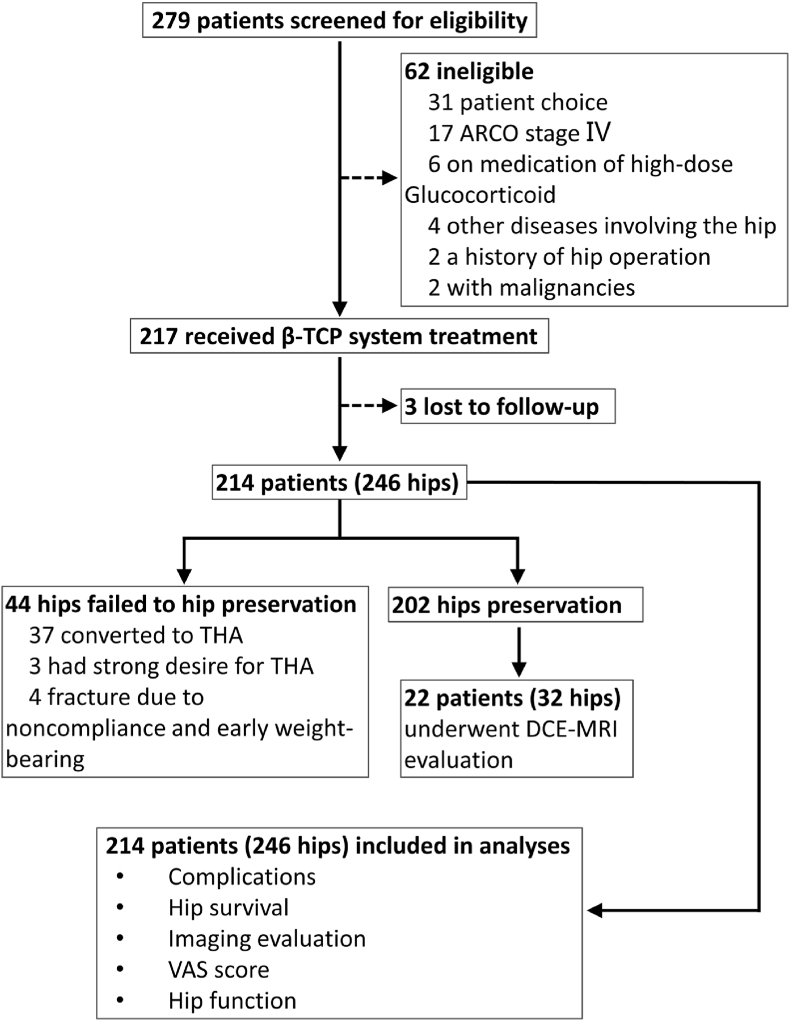
Profile of the clinical trial.

A total of 214 eligible and assessable patients with 246 hips were enrolled in the clinical study (Fig. 6), including 62 females and 152 males, with a median age of 43 years (range, 17–65 years). The causes of ANFH were alcohol abuse (103 hips, 41.87%), corticosteroid application (81 hips, 32.93%), trauma (16 hips, 6.5%), and idiopathic (46 hips, 18.7%). According to the ARCO staging system, 157 (63.82%) hips were in stage Ⅱ, and the remaining 89 (36.18%) were in stage Ⅲ. All patients were clinically symptomatic, with a VAS of 4.57 ± 1.56 and an HHS of 57.95 ± 12.06. The baseline characteristics of the included patients are shown in Table 1. The mean follow-up period was 42.79 ± 12.89 months (range, 3–60 months).

**Table 1:** Baseline characteristics of the enrolled patients (n = 214 patients, 246 hips).

	n (%)
**Gender**	
Male	152 (71.03%)
Female	62 (28.97%)
**Age**	
Median (range)/years	43 (17–65)
**Body mass idex (BMI)**	
Mean ± SD/kg/m^2^	23.60 ± 2.86
**Etiology (hips)**	
Alcohol abuse	103 (41.87%)
Corticosteroid application	81 (32.93%)
Trauma	16 (6.5%)
Idiopathic	46 (18.7%)
**ARCO stage (hips)**	
Ⅱ A	24 (9.8%)
Ⅱ B	62 (25.2%)
Ⅱ C	71 (28.9%)
Ⅲ A	42 (17.1%)
Ⅲ B	28 (11.4%)
Ⅲ C	19 (7.7%)
**Left or right (hips)**	
Left	117 (47.56%)
Right	129 (52.44%)
**Preoperative HHS (hips)**	
Mean ± SD	57.95 ± 12.06
**Disease course**	
Mean ± SD/months	6.52 ± 2.81
**Comorbidities**	
Hypertension	26 (12.15%)
Systemic lupus erythematosus	21 (9.81%)
Type Ⅱ diabetes	17 (7.94%)
Immune nephropathy	12 (5.61%)
Dermatomyositis	6 (2.80%)
Bronchial asthma	6 (2.80%)
Psoriasis	2 (0.93%)
None	124 (57.94%)
**Preoperative VAS score**	
Mean ± SD	4.57 ± 1.56

#### 3.5.2 Intraoperative and postoperative complications

The procedure was successful in all patients, with a mean operative duration of 65.24 ± 21.8 min and a mean intra-operative blood loss of 50.84 ± 19.33 mL. One patient experienced dyspnea during the operation and was suspected of having a pulmonary air embolism due to rapid bone grafting. The situation was stabilized after resuscitation, and the patient was returned to surgery. Six patients (2.8%) experienced postoperative problems, including four femoral neck fractures due to noncompliance with medical recommendations on protective weight-bearing, and two wound infections, both of which were resolved.

#### 3.5.3 Survival of the femoral head

**Fig. 7:**
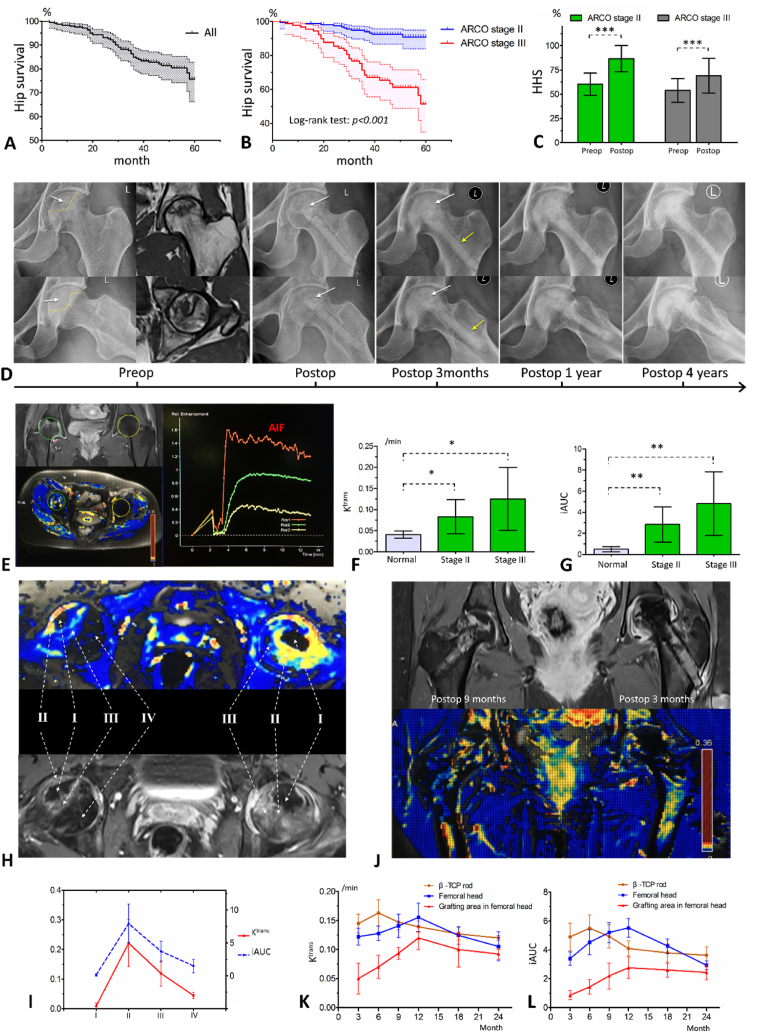
Clinical trial results. A. Kaplan-Meier survival curve of operated femoral heads. B. Kaplan-Meier survival curves of operated femoral heads in ARCO stage Ⅱ or Ⅲ. C. The preoperative and postoperative HHSs of operated hips in ARCO stage Ⅱ or Ⅲ. D. A patient with ARCO stage Ⅲ ANFH caused by corticosteroid application. X-ray films show the degradation of the implanted β-TCP scaffold and the substitute of newly formed bone. Four years after surgery, the necrotic femoral head was completely recovered. The white arrows indicate the necrotic area in femoral head. The yellow arrows indicate that the gap between implanted β-TCP rod and host bone was gradually healed. E. DCE-MRI examination comparing necrotic and normal femoral head. On the left is the delineation of the region of interest (ROI) and the cloud diagram of K^trans^ values, in which the green circle indicates a necrotic femoral head in ARCO stage Ⅱ, and the yellow circle indicates a normal femoral head. On the right are the SI-time curves of ROIs. AIF indicates the arterial input function. F. K^trans^ values of normal and necrotic femoral heads in ARCO stages Ⅱ and Ⅲ. G. iAUC values of normal and necrotic femoral heads in ARCO stages Ⅱ and Ⅲ. H. Blood perfusions in different zones of the necrotic femoral head. Ⅰ, complete necrosis with no blood perfusion; Ⅱ, repair zone with high perfusion; Ⅲ, compensatory zone with relatively high perfusion; and Ⅳ, normal bone. I. K^trans^ and iAUC of different zones in the necrotic femoral head; J. DCE-MRI examination after surgery. The left and right femoral head were nine and three months postoperatively, respectively. K. Changes in K^trans^ values after implantation of the β-TCP system. L. Changes of iAUC values after implantation of the β-TCP system. *p < 0.05, **p < 0.01, ***p < 0.001.

At the final follow-up, the survival rate of the femoral head was 82.1% (202/246). A total of 44 hips (17.89%) had failures in hip preservation, of which 37 were converted to THA as the disease progressed; four were due to fracture, and three had insufficient hip functions and a strong desire for THA. The 1-year, 3-year and 5-year survival rates calculated based on Kaplan-Meier analysis were 98% (95% confidence interval (CI): 96.2%–99.7%), 84.9% (95% CI: 80.2%–89.6%), and 75.6% (95% CI: 67.4%–83.8%), respectively (Fig. 7A). Patients with ARCO stage Ⅱ showed significantly greater femoral head survival than those with stage Ⅲ (log-rank test, p < 0.001) (Fig. 7B).

#### 3.5.4 Hip function evaluation

The mean HHS improved from 57.95 ± 12.06 (range, 22.2–85.0) preoperatively to 80.26 ± 17.43 (range, 23.5–100.0) at the last follow-up (p < 0.001), with 87 (35.4%) graded excellent, 76 (30.9%) good, 22 (8.9%) fair and 61 (24.8%) poor. The mean HHS for ARCO stages Ⅱ and Ⅲ were 86.6 ± 13.5 and 69.0 ± 18.0, respectively, which were significantly different (p < 0.001) (Fig. 7C). A total of 87% (n = 214) of the patients had partial or complete recovery of hip function, with increases in HHSs of 27.2 ± 11.9.

#### 3.5.5 Imaging evaluation

Material degradation and bone tissue regeneration were detected radiologically by X-rays and CT scans (Fig. 7D). Among the 246 hips, 185 (75.2%) were radiologically stable or better, and 61 (24.8%) showed worse imaging results. New collapse or necrosis expansion occurred in 12 (7.6%) hips in stage Ⅱ, and 24 (27.0%) in stage Ⅲ (p < 0.05).

#### 3.5.6 Reperfusion of the femoral head evaluated by DCE-MRI

DCE-MRI examination was performed on 22 patients with 32 hips. The entire necrotic femoral head presented a state of hyperperfusion, with iAUC (p = 0.013) and K^trans^ (p = 0.021) values significantly higher than those in the normal femoral head (Fig. 7E, F, and 7G). However, such an increase in blood perfusion was mainly concentrated around the necrotic areas, with essentially no blood perfusion occurring within the necrotic area (Fig. 7H and I). It is suggested that there is a challenge to irrigate the necrotic region due to the barriers of necrotic tissue and sclerotic band. After removing the necrotic tissues and implanting the β-TCP system, the blood perfusion in the β-TCP rod increased rapidly within three months, and over time, the regenerative blood flow was directed to the graft location in the femoral head (Fig. 7J, K and 7L). After surgery, the iAUC and K^trans^ values of the affected femoral head steadily increased and peaked in the 12th month. Later, blood perfusion gradually asymptotized to the level of the normal femoral head (Fig. 7K and L), signifying that the repair of the necrotic bone and vascular maturation was fcomplete.

#### 3.5.7 VAS pain scores

The mean VAS score of patients at the time of the last follow-up was 1.35 ± 1.30 (range, 0–8), significantly decreased from the baseline values (4.57 ± 1.56, range 1–8, p < 0.001). Patients with ARCO stage Ⅲ disease had significantly higher pain scores than those with ARCO stage Ⅱ (4.97 ± 1.53 vs. 4.33 ± 1.54, p = 0.002), but after treatment, there was no significant difference (p = 0.148). The results indicated significant relief of the hip pain caused by ANFH.

#### 3.5.8 Factors influencing therapeutic effect

The variables of age, gender, BMI, disease course, operated side, etiology, ARCO stage, comorbidities, preoperative VAS scores, and preoperative HSSs were entered into a multivariate linear regression analysis investigating clinical efficacy based on hip function (HHS). Poor therapeutic effect were associated with old age (p < 0.01), late ARCO stage (p < 0.01), and low preoperative HHSs (p < 0.01), but not with gender (p = 0.353), operated side (p = 0.732), disease course (p = 0.320), BMI (p = 0.285), etiology (p = 0.707), preoperative VAS (p = 0.880), or comorbidities (p = 0.379) (Table S1, Supporting Information).

## 4 Discussion

In this study, we estabished a β-TCP system for the hip-preserving treatment of ANFH, which bio-adaptively reconstructed the necrotic femoral head. The bioadaptive tissue regeneration (neovascularization and new bone formation) and mechanical adaptation of this system were validated through animal experiments, mechanical tests, computer simulations, and a clinical trial. The theoretical foundations for using the β-TCP system as a hip-preserving therapy for ANFH include the following. (1) Breaking barriers. Necrotic lesions were removed, and the sclerotic zone was opened, creating a bioactive healing environment for bone regeneration. (2) Bioadaptability. The degradable implants actively interacted with the host microenvironment and promoted tissue regeneration. (3) Reviving „no man's land“. Porous β-TCP scaffolds with ideal interconnected architectures were proved to have superior vascularization characteristics, making them a bridge to direct newly generated blood vessels into the femoral head, reviving the previously dormant necrotic regions. (4) Refreshing femoral head. The newly produced bone progressively filled and replaced the porous scaffold after angiogenesis, finally establishing healthy bone tissue within the femoral head. (5) Recovering mechanical properties. The bio-adaptive reconstruction resulted in the continuous recovery of mechanical function during the process of material degradation and bone regeneration, which avoided femoral head collapse. These above bases disrupted the pathological process of ischemia and necrosis of the femoral head, thereby curing ANFH.

Essentially, femoral head blood supply obstruction finally causes ANFH. Thus, studies have concentrated on employing vascularized bone grafts, such as fibula grafts [37], iliac bone flaps [38], and greater trochanteric bone flaps [39], to reestablish blood flow to the femoral head. Although these methods have clinical results superior to osteotomies, tantalum rods, and non-vascularized bone grafting, they are associated with severe surgical trauma, graft inactivation risk, and challenging donor-site morbidity [40]. Another potential technique for treating ANFH is the use of synthetic biomaterials, which avoid the drawbacks of autologous bone grafting [41,42]. Nevertheless, bone substitutes that simply focus on anatomical recovery or mechanical support were demonstrated to inadequately treat ANFH due to the lack of bioadaptability. For example, stress shielding of non-degradable metallic materials (tantalum rod, etc.) may result in implant loosening over time [43], and the rapid degradation of the calcium-sulfate scaffold may result in the resporption of newly formed bone tissue [44]. The treatment of ANFH, should primarily be a combination of mechanical reconstrustion and biological repair, with revascularization of the necrotic femoral head the most important.

Several strategies for improving the vascularization of synthetic scaffolds have been proposed, including angiogenic factors administration [45], surgical pre-vascularization [25], and the addition of vascular-seeded cells [46]. However, the porosity of the scaffold is the most critical moderator in determining vascularization, which plays a key role in delivering oxygen and nutrients and eliminating cell metabolic waste. It determines the direction of angiogenesis, which predicts the depth of vascularization. It is generally believed that higher porosity and internal connectivity between pores favor vascularization. However, increased porosity will exponentially reduce mechanical strength and accelerate degradation, which is detrimental to bone regeneration. After a series of studies, we created an ideal porous β-TCP scaffold with high homogeneity and connectivity to counteract the imbalance between vascularization and mechanical loss (macropores 400–500 m, interconnection 110–120 m, and porosity: ≈75%). Each macropore and the four surrounding interconnected pores in this scaffold formed an effective vascular/bone repair unit, and these units were joined to create a useful vascular/bone network (Fig. S7, Supporting Information). In this study, we visualized and quantitatively assessed vascularization within the β-TCP scaffold using a novel angiography method. Our results revealed that this β-TCP scaffold could be rapidly and adequately vascularized after implantation. Further, the newly formed inner vessels could be directed into the encapsulated barren areas where communication with surrounding tissues was blocked. This discovery has evolved into the cornerstone for using the bioactive β-TCP system to treat ANFH. Our clinical study also confirmed the property of porous β-TCP scaffolds to direct abundant blood supply around the necrotic regions to the implanted regions, enabling reperfusion of the ischemic femoral head (Fig. 7H-L, Fig. S8, Supporting Information).

Another concern for using the β-TCP system in the treatment of ANFH is mechanical strength due to its inherent brittleness. Full mechanical recovery did not occur in this study during the initial post-implantation stage. Even with the addition of dense granules, the implantation of the β-TCP system could only partially restore mechanical support in the immediate postoperative period. However, it is important to emphasize that the mechanical characteristics of the femoral head and neck gradually increased and eventually reverted to normal during tissue regeneration. Unlike other tissues, bone tissue regeneration must be accompanied by adaptive mechanical reconstruction to be meaningful. Schematically, bone tissue formation necessitates both biochemical reactions and stress stimulation. The degradation products of β-TCP (calcium and phosphorus), together with the recruited cytokines and seed cells, formed a repair microenvironment that promoted osteogenesis. On the other hand, mechanically suitable β-TCP permitted stress to be transmitted to osteocytes, thus promoting the stability of nascent bone tissue. Throughout the process of β-TCP degradation and tissue regeneration, the mechanical properties of the reconstructed regions dynamically and progressively adapted to a native living system. We discovered that the trabeculae of the new bone were remodeled along the stress direction and integrated with the trabeculae of the host bone during the process of tissue reconstruction, and eventually, the normal stress microarchitecture was restored. Some previous research concentrated exclusively on the rigid support of the femoral head, ignoring tissue regeneration and mechanical adaptation. However, all these attempts including the use of tantalum rod, ultimately failed [43].

Our clinical results revealed a promising effects of the β-TCP system in the treatment of ANFH. The hip survival rate was 82.1% for ARCO stages Ⅱ– Ⅲ disease, which was superior to the traditional method of core decompression (Table 2) [[4], [5], [6],^47^]**.** In a meta-analysis of 20 studies involving 1323 cases who underwent core decompression, the hip survival rate was 66% during 54.3 months of median follow-up [4]. The clinical results in our study were also superior to other non-vascularized bone substitute materials, including tantalum rod implantation [48,49], CaSO4–CaPO4 [50], demineralized bone matrix [51,52], allogeneic bone [[53], [54], [55]], and so on. In recent years, a variety of artificial materials have been studied for treating ANFH. However, the benefits were mostly observed in experimental studies, and only a few were confirmed in clinical studies. The most important reason is the lack of bioadaptability, which makes reactivating the femoral head difficult. Cell-based therapies, such as compounding with scaffold materials, were also introduced to treat ANFH and produced promising clinical outcomes, making them more suitable for adjunctive ANFH therapy [4,47,56,57]. To enrich the seed cells for angiogenesis and osteogenesis, we allowed healthy bone marrow collected during tunneling to mix into the β-TCP scaffold before transplanting it into the defects. Autologous bone grafting, considered the gold standard for bone defect repair, has shown good clinical results, whether implanted via core decompression or the lighting method since fresh autologous bone is enriched with the necessary conditions for tissue regeneration. The rate of hip survival after treatment with the β-TCP system in our study (82.1%) was comparable to that of autologous bone grafting (82%–86.7%) [[4], [5], [6],^8^,^58^], and more crucially, it avoided secondary injury and possible postoperative complications caused by bone harvesting. However, our outcomes, however, were inferior to those of vascularized bone grafts [7,38,59], which restore blood supply and commence healing immediately after surgery.

**Table 2:** The clinical outcomes of different hip preservation methods for ANFH.

Study	Article type	Sample size(No. of hips)	Stage of disease	Age	Implants or procedures	Median follow-up (months)	Hip survival (%)	HHS
Core decompression								
K. Hua, 2019	Meta-analysis	1323	ARCO Ⅰ-Ⅲ;	37.72 (12–85)	Traditional core decompression	54.3	66%	/
Q. Wang, 2020	Retrospective study	59	ARCO Ⅱ	39.1 ± 10.2	Traditional core decompression	48	69.5%	80.2; 7.3 improved
S. Lakshminarayana, 2019	Observational study	36	Fiact Ⅰ-Ⅱ	30.07 (18–48)	Traditional core decompression	53.5	/	77; 29 improved
J. S. Kang, 2018	Retrospective study	53	ARCO Ⅰ-Ⅳ	47.3 ± 9.7	Traditional core decompression	51.36	51%	/
**Core decompression combined with cell-based therapy**								
M. Grassi, 2020	Prospective study	30	Ficat Ⅰ-ⅡB	42 (23–60)	Traditional core decompression; PRP	60	53%	84; 20 improved
K. Hua, 2019	Meta-analysis	372	ARCO Ⅰ-Ⅲ	37.72 (12–85)	Traditional core decompression; Bone marrow	54.3 (2–228)	84%	/
J. S. Kang, 2018	Retrospective study	53	ARCO Ⅰ-Ⅳ	46.0 ± 9.3	Traditional core decompression; BMMSC	51.36	71.7%	/
Y. W. Lim, 2013	Retrospective study	128	Ficat Ⅰ-Ⅲ	36.3 ± 9.7	Multiple drilling core decompression; BMMSC	87 (8–134)	55.5%	/
**Bone substitute materials implantation**								
S. Landgraeber, 2017	Prospective study	31	ARCO Ⅱ	42.9 (21–60)	Calcium sulfate (CaSO4)-calcium phosphate (CaPO4)	30.06 (12–43.3)	75.9%	83.78; 10.13 improved
B. L. Wang, 2010	Prospective study	138	ARCO ⅡA-ⅢA	32.36 (17–54)	Demineralized bone matrix (DBM) and auto-iliac bone	25.37 (7–42)	ⅡA-ⅡB: 85%; ⅡC-ⅢA: 60%	79; 17 improved
J. E. Hsu, 2011	Prospective study	62	Steinberg Ⅰ-Ⅱ	40.6 (20–65)	Grafton Demineralized Bone Matrix (Osteotech, Eatontown)	46.3 (24–107)	62.9%	/
S. B. Kizer, 2006	Retrospective study	80	Ficat Ⅰ-Ⅳ	36 ± 13.2	Allogeneic cortical strut grafts (tibia/fibular)	108 (48–204)	59%	/
B. F. Wei, 2011	Retrospective study	223	ARCO Ⅱ-ⅡA	33.5 (19–54)	Allogeneic fibular	24	81%	/
Y. Zeng, 2015	Retrospective study	18	ARCO ⅡB-ⅡC	40.7 (22–49)	Allogeneic bone	53.3 (20–107)	78%	83.8; 22.2 improved
**Tantalum rod implantation**								
Y. Liu, 2015	Prospective study	59	Steinberg Ⅰ-Ⅳa	43 (21–70)	Porous tantalum rod	60	72.49%	78; 19 improved
Y. Zhang, 2021	Retrospective study	52	ARCO Ⅰ-Ⅱ	40.1 ± 9.3	Porous tantalum rod	85.7 (60–132)	52.9%; 6-year: 60%	69.7; 3.7 reduced
**Non-vascularized autograft implantation**								
K. Hua, 2019	Meta-analysis	427	ARCO Ⅰ-Ⅲ	37.72 (12–85)	Traditional core decompression; Autologous bone	54.3 (2–228)	82%	/
S. Lakshminarayana, 2019	Observational study	40	Ficat Ⅰ-Ⅱ	30.07 (18–48)	Traditional core decompression; non-vascularized fibular graft	53.5 (44–63)	/	71.5; 8.5 improved
Q. Wang, 2020	Retrospective study	66	ARCO Ⅱ	38.1 ± 10.0	Light bulb technique; Autologous bone	48	84.8%	83.1; 9.4 improved
C. Yildiz, 2017	Retrospective study	28	Steinberg Ⅰ-Ⅳ	34 (22–50)	Light bulb technique; Autologous bone	52.6 (24–80)	82.1%	74.33; 21.67 improved
D. Li, 2017	Retrospective study	83	ARCO Ⅱ	38.2 ± 8.2	Light bulb technique; Autologous bone	36 (32–44)	86.7%	86.5; 14.1 improved
**Vascularized bone grafting**								
L. Cao, 2017	Prospective study (RCT)	21	ARCO Ⅰ-ⅢB	31 ± 6	Free vascularized fibular grafting	36	95.2%	82
H. Xie, 2019	Retrospective study	1006	Ficat Ⅱ-Ⅳ	38 (18–55)	Vascularized iliac bone grafting	60	88.7%	87.43; 21.01 improved
D. Zhao, 2017	Retrospective study	2190	Ficat Ⅱ-Ⅳ	43.15 ± 9.14	Pedicled iliac bone flap transfer	144 (60–300)	90.1%	83.63; 17.09 improved
**β-TCP system in this study**								
Y. Lu et al.	Prospective study (multi-center)	246	ARCO Ⅱ-Ⅲ	43 (17–65)	β-TCP system	42.79 ± 12.89	82.1%	80.26; 22.31 improved

Notes: ARCO, Association Research Circulation Osseous; PRP, platelet-rich plasma; BMMSC, bone marrow mesenchymal stem cell.

Many factors have been reported to be associated with the prognosis of ANFH. Our clinical results suggested that patients with older age, later stage, and lower preoperative HHS had worse clinical outcomes. We confirmed that employing the β-TCP system for treating ANFH in ARCO stage Ⅱ was much superior to stage Ⅲ in terms of hip survival, imaging outcomes, and hip functional assessment. Therefore, we strongly recommend this technique for young patients with ANFH who are in the pre-collapse stage. During clinical application, some modified methods based on the β-TCP system were developed for specific types of ANFH. For example, small β-TCP rod implantation assisted by computer navigation was used for hip preservation in ANFH patients with small lesions, as well as secondary hip preservation for disease progression after previous β-TCP system treatment (Fig. S9, Supporting Information). Those methods should be further explored and verified.

This study had some limitations. First, this was a clinical translational study and did not demonstrate a specific molecular mechanism of vascularization in the β-TCP scaffold. Second, in animal experiments, we prepared a semi-encapsulated β-TCP rod to simulate the local ischemic bone regeneration environment, which represents the ultimate pathological feature of ANFH. However, such a simulation does not fully reflect the dynamic process of ANFH. Therefore, it is necessary to further validate the findings of this study in a large animal model of ANFH. Third, the novel method for vascular visualization can only be used in animal experiments, but not for the evaluation of the vascularization of human femoral head. Finally, although a multi-center prospective trial was conducted, the sample size was relatively small, and long-term results were lacking. Therefore, further clinical trials are required.

## 5 Conclusion

To summarize, we created a bioceramic system using β-TCP scaffolds for ANFH reconstruction, which was fully consistent with the parameters of bioadaptability and was clinicaly proven to be effective. The optimal interconnected microstructure of porous β-TCP stimulated adaptive neovascularization and concurrent bone regeneration, guiding the surrounding active environment to facilitate necrotic femoral head restoration. Along with material degradation and bone regeneration, the mechanical characteristics of the femoral head were dynamically and adaptively improved and eventually restored to normal. When transferred to clinical trials, β- TCP demonstrated a potential clinical benefit for ANFH, particularly in young individuals with early-stage disease.

## Funding statement

This work was funded by 10.13039/501100001809the National Natural Science Foundation of China (NSFC) (No.32000960), National key research and development program (2021YFB3800800), and Shanghai "Science and Technology Innovation Action Plan" Biomedical Support Project (22S31906500).

## Data availability statement

Some or all data during the study are available from the corresponding author by request.

## Ethics approval statement

The Animal experiment was conformed to the animal welfare act and approved by the Ethics Committee of Xijing Hospital. The clinical trial was approved by the institutional review board at participating institution (No.KY20162052-1) and registered at the Chinese Clinical Trial Registry (ChiCTR-ONh-16008989). All patients were consented and signed an informed consent form.

## Patient consent statement

I confirm that I have obtained all consents required by applicable law for the publication of any personal details or images of patients, research subjects or other individuals that are used in the materials submitted to KeAi. I have retained a written copy of all such consents and I agree to provide KeAi with copies of the consents or evidence that such consents have been obtained if requested by KeAi.

## Submission declaration

The work described has not been published previously.

## CRediT authorship contribution statement

**Yajie Lu:** Conceptualization, Methodology, Formal analysis, Funding acquisition, Writing – original draft. **Xiantao Chen:** Methodology, Investigation, Formal analysis. **Xiao Lu:** Methodology, Validation, Formal analysis. **Changning Sun:** Methodology, Validation. **Minghui Li:** Formal analysis, Investigation, Visualization. **Guojing Chen:** Resources, Investigation. **Zuoyao Long:** Formal analysis, Investigation. **Yuan Gao:** Resources, Data curation. **Haoqiang Zhang:** Formal analysis. **Mengquan Huang:** Investigation. **Chuanlei Ji:** Methodology. **Hongbin Fan:** Investigation. **Dong Liu:** Investigation. **Yuewen Hao:** Methodology, Formal analysis. **Hong Wang:** Methodology, Formal analysis. **Leilei Zhang:** Investigation. **Hongmei Zhang:** Formal analysis. **Jianxi Lu:** Conceptualization, Resources, Supervision. **Zhen Wang:** Conceptualization, Methodology, Supervision, Writing – review & editing. **Jing Li:** Writing – review & editing, Supervision.

## Declaration of competing interest

None.
