# Supplementary material for: Reconstructing avascular necrotic femoral head through a bioactive β-TCP system: From design to application
Source: Bioact Mater. 2023 Jun 26;28:495–510. doi: 10.1016/j.bioactmat.2023.06.008 (PMC10318430; doi:10.1016/j.bioactmat.2023.06.008)
Supplement: Multimedia component 1 [file mmc1.docx]

Supplementary Information

**Supplementary information – Figure S1**


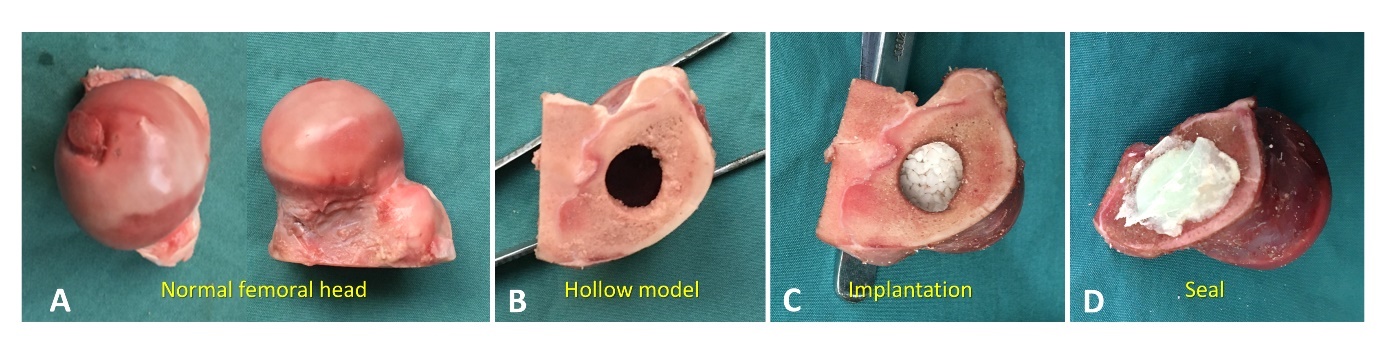


**Figure S1** Implantation models in pig femoral head for compressive tests. A. Normal femoral head. B. Femoral head with a central cavity. C. Femoral head implanted with porous, dense, or mixed β-TCP granules. D. The opening for implantation was sealed with bone cement.

**Supplementary information – Figure S2**


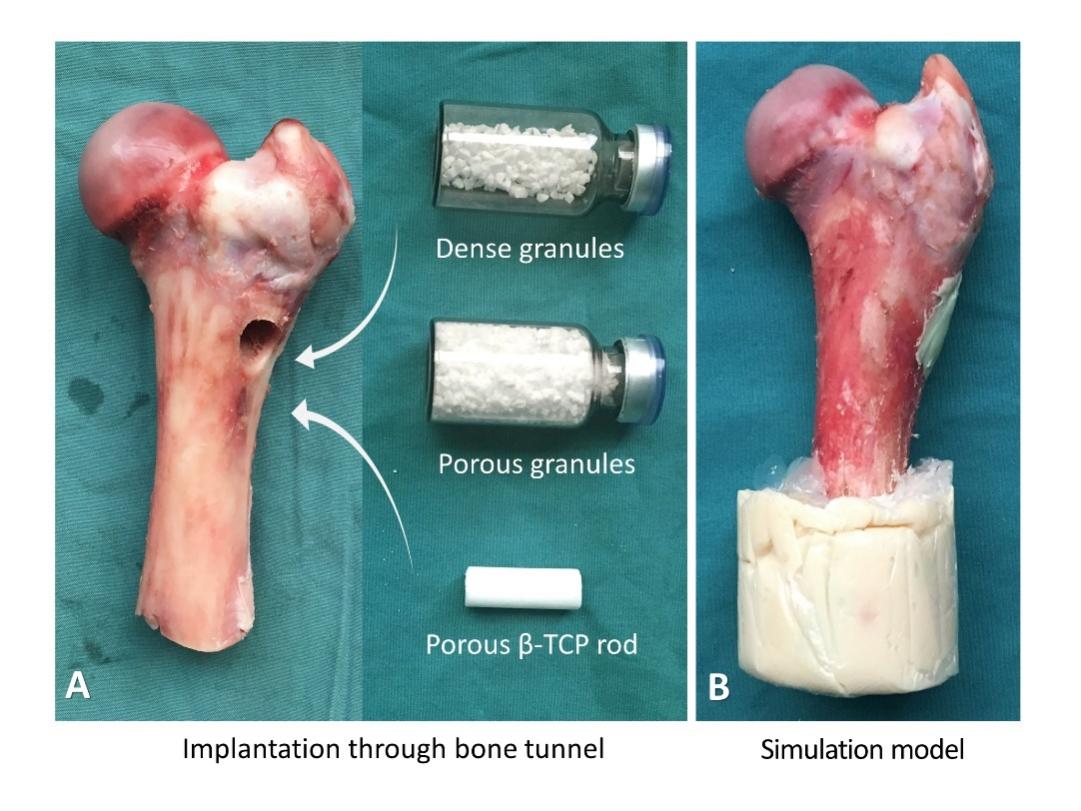


**Figure S2** Surgical model in pig proximal femur for mechanical test in standing posture. A. After core decompression and simulated curettage of necrosis in the femoral head, the dense and porous β-TCP were compacted into the cavity, followed by implantation of a porous β-TCP rod. B. The femoral specimens were anchored at 15 ° adduction and 10 ° internal rotation to simulate the single-legged stance position.

**Supplementary information – Figure S3**


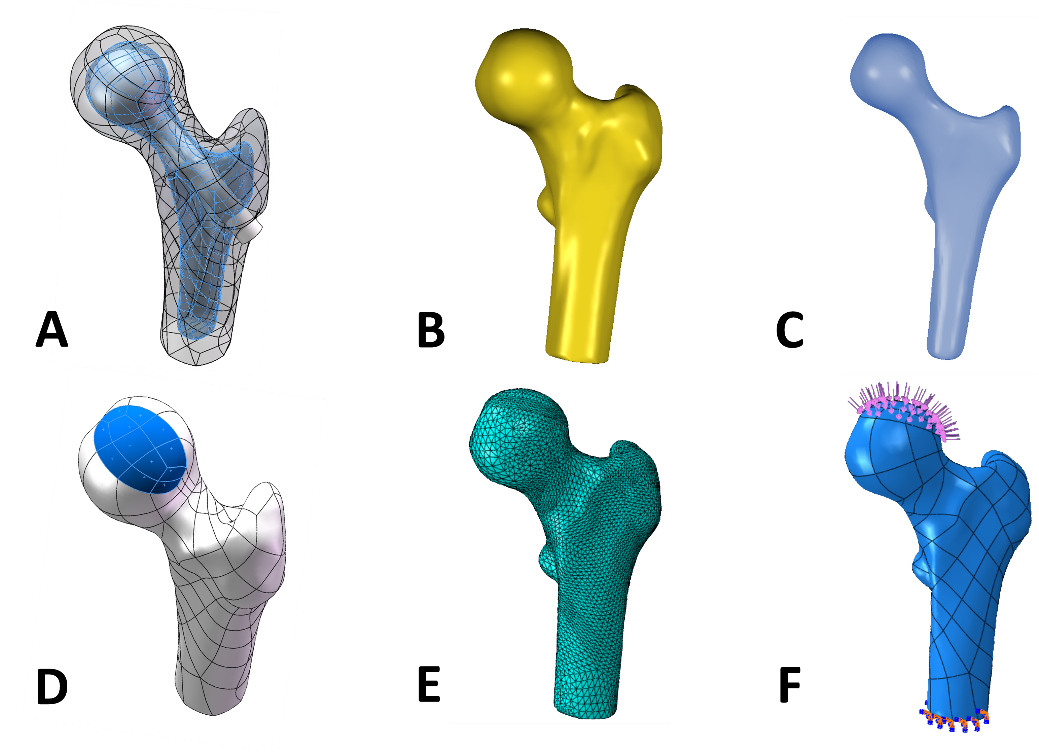


**Figure S3** The models of three-dimensional finite elements analysis. A Simulated surgical model of core decompression, necrotic curettage, and the implantation of a β-TCP scaffold. B. Cortical bone model; C. Cancellous bone model. D. Weight-bearing area of femoral head surface. E. Mesh of the finite element model, a total of 375471 elements were generated. F. A load of 700N was applied on the weight-bearing area of femoral head surface, with the distal end of femur restrained in all directions.

**Supplementary information – Figure S4**


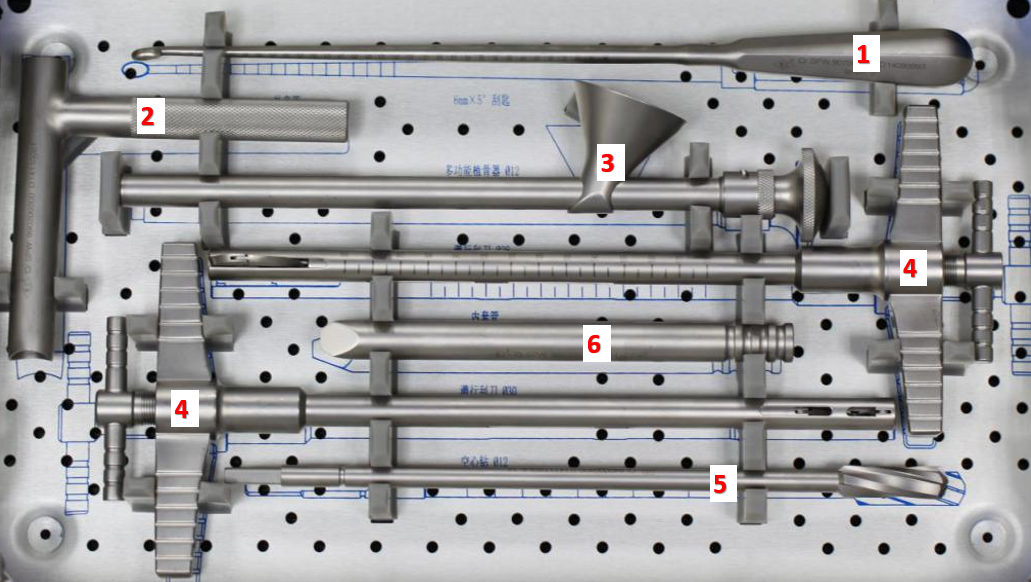


**Figure S4** The customized surgical instruments for ANFH. 1. surgical spoon curette. 2. guide cannula; 3. graft delivery device. 4. stealth scraper. 5. cannulated hollow reamer. 6. guide core.

**Supplementary information – Figure S5**


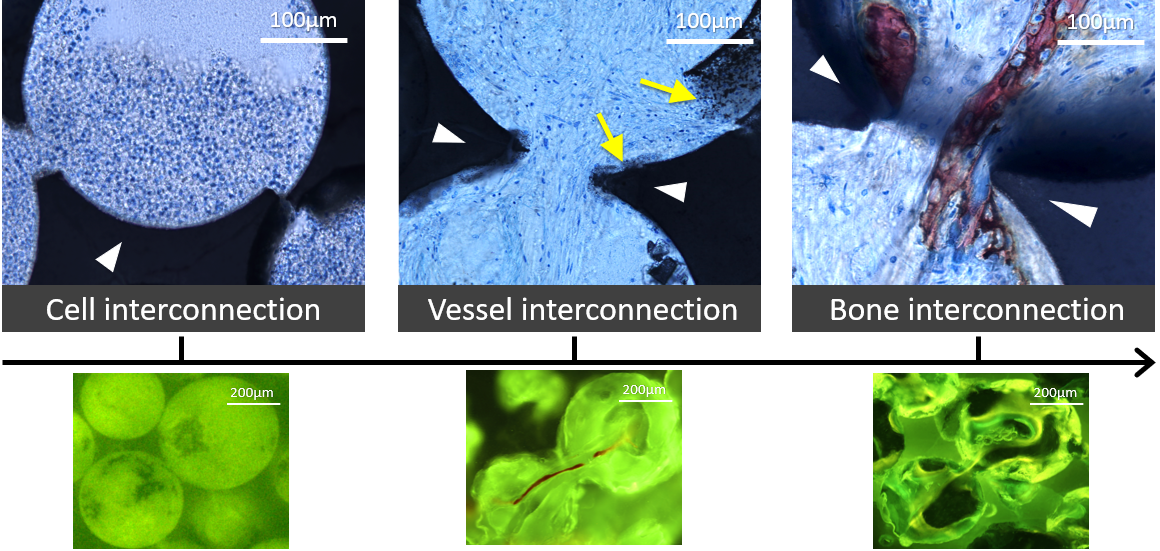


**Figure S5** Three stages of bone regeneration in the porous β-TCP scaffold. Left to right: cell interconnection, blood vessel inter connection, and bone interconnection. The white triangles indicate the β-TCP scaffold, the yellow arrows indicate the partly degraded material granules.

**Supplementary information – Figure S6**


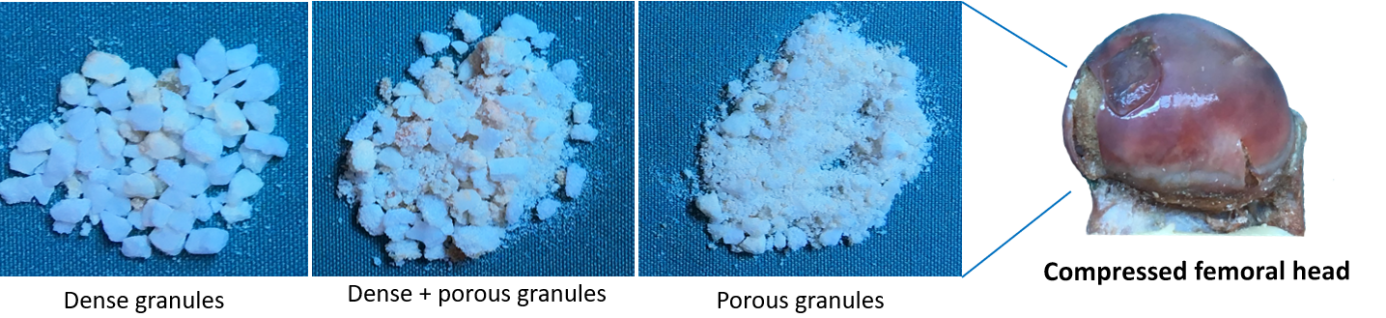


**Figure S6** Gross view of implanted dense granules, mixed granules, and porous granules after femoral head compressive test.

**Supplementary information – Figure S7**

**
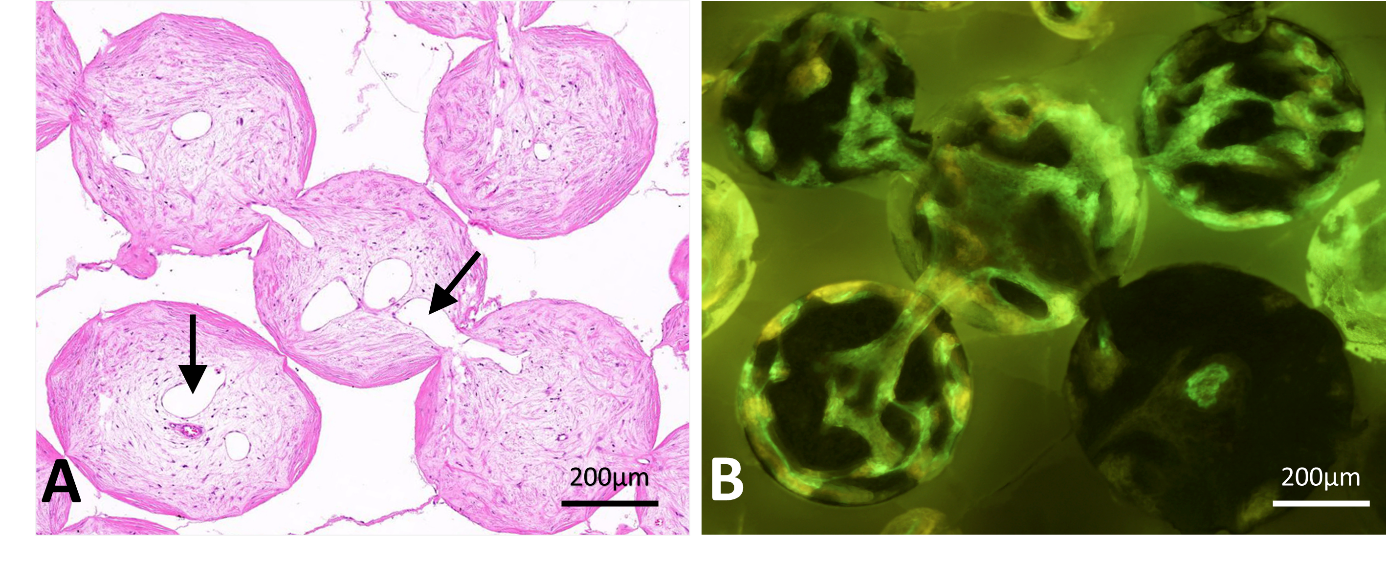
**

**Figure S7** Histological confirmation of the “basic repair unit” in β-TCP scaffold. A. Histological confirmation of the repair unit (H.E. Staining): tissues and blood vessels in the macropores were connected through interconnected holes. The black arrows indicate blood vessels. B. Fluorescence confirmation of the repair unit (tetracycline-calcein labeling): the newly formed bone tissue in each macropore were connected to each other through interconnected holes.

**Supplementary information – Figure S8**


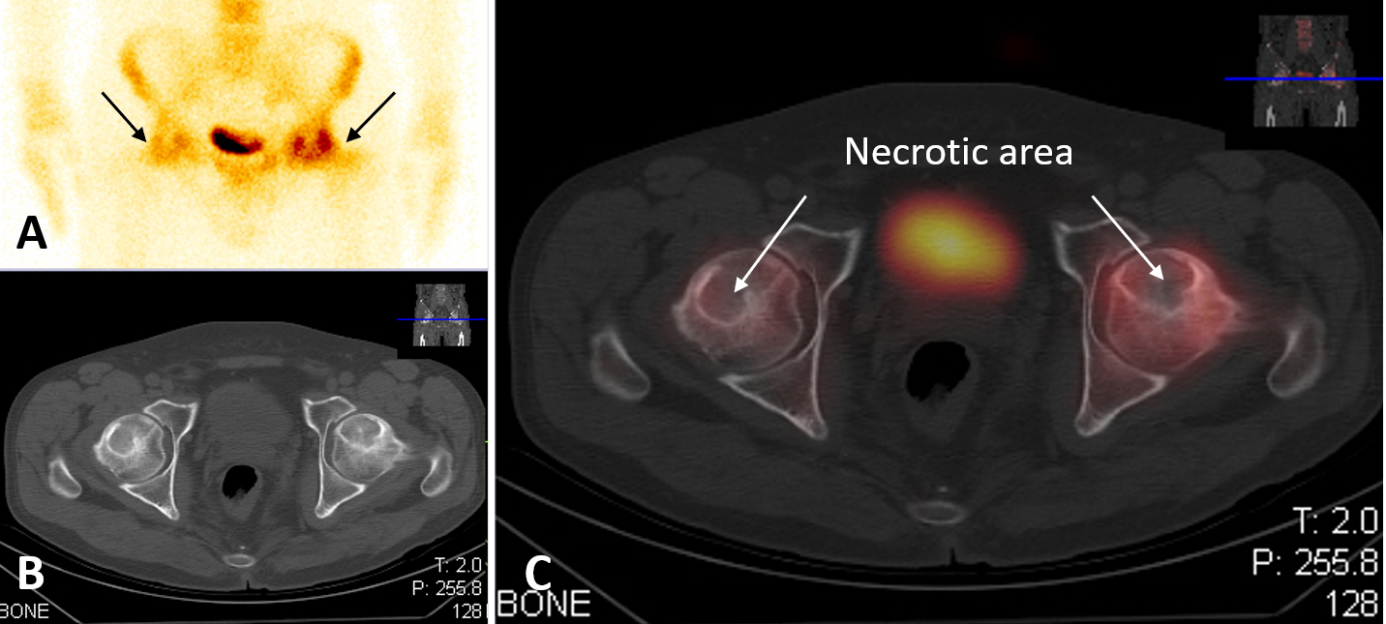


**Figure S8** The SPECT/CT results of ANFH. A. SPECT image shows a patient with with bilateral femoral head necrosis; B Axial CT image shows the necrotic lesions of ANFH; C. SPECT/CT image shows active bone metabolic activity around the necrotic area.

**Supplementary information – Figure S9**


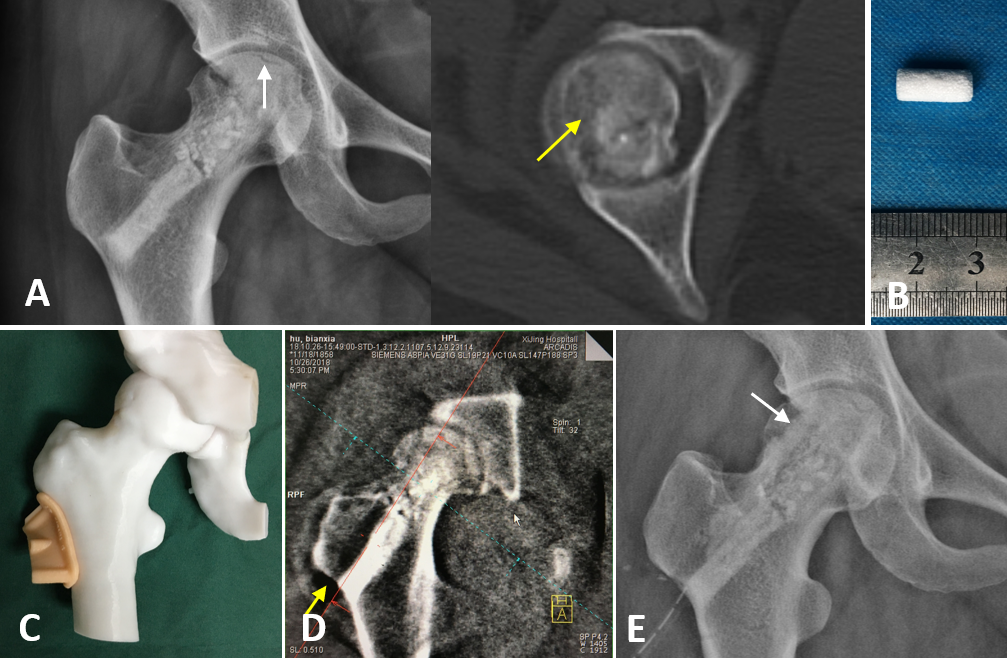


**Figure S9** Small β-TCP rod implantation technique used for secondary hip preservation. A. A patient who had undergone β-TCP system for hip-preservation had disease progression 1 year after surgery. The white arrow indicates the fractures of the subchondral bone, and the yellow arrow indicates small lesions in femoral head. B. The small porous β-TCP rod (diameter, 5mm). C. Design of the guide plate for the surgical channel. D. Precise localization of necrotic lesions and implantation of small β-TCP rods with the aid of computer navigation. The yellow arrow indicates the small surgical channel through femoral neck. E. Postoperative X-ray film. The white arrow indicates the position of small β-TCP rod.

**Supplementary information – Table S1**

**Table S1** Multivariate analysis of prognostic factors for hip functional results.

| **Factors** | **Variables** | **β** | **95% CI (low to high)** | **P value** |
| --- | --- | --- | --- | --- |
| **Age^*^** | Continuous data (17-65) | -0.210 | -0.470 to -0.138 | <0.01 |
| **Gender** | Male | / | / |  |
|  | Female | -0.061 | -7.216 to 2.583 | 0.353 |
| **Operated side** | Right | / | / |  |
|  | Left | 0.018 | -2.977 to 4.231 | 0.732 |
| **Disease Course** | Continuous data (1.5-16) | 0.053 | -0.323 to 0.984 | 0.320 |
| **BMI** | Continuous data (15.35-27.89) | 0.057 | -0.289 to 0.977 | 0.285 |
| **Etiology** | Corticosteroid application | / | / |  |
|  | Alcohol abuse | -0.030 | -6.602 to 4.481 | 0.707 |
|  | Trauma | 0.040 | -5.019 to 10.682 | 0.478 |
|  | Idiopathic | -0.054 | -8.051 to 3.195 | 0.396 |
| **ARCO stage ^*^** | Ranked data (1-6)^†^ | -0.456 | -7.135 to -4.283 | <0.01 |
| **Pre HHS^*^** | Continuous data (22.2-85) | 0.206 | 0.133 to 0.461 | <0.01 |
| **Pre VAS** | Continuous data (1-8) | -0.008 | -1.331 to 1.142 | 0.880 |
| **Comorbidities** | None | / | / |  |
|  | Yes | -.047 | -5.442 to 2.080 | 0.379 |

Notes:*p<0.01; †, ranked data for ARCO stage: IIA(1), IIB(2), IIC(3), IIIA(4), IIIB(5), IIIC(6).

**Supplementary information – Table S2**

**Table S2** Assigned material properties for the FEA model.

| **Material** | **Young’s modulus (Mpa)** | **Poisson’s ratio** |
| --- | --- | --- |
| Cortical bone | 10000 | 0.3 |
| Cancellous bone | 1590 | 0.3 |
| Porous β-TCP granules | 440 | 0.3 |
| Dense β-TCP granules | 1548 | 0.3 |
| Porous β-TCP rod | 440 | 0.3 |
| Mixed β-TCP granules ^*†^ | 756 | 0.3 |
| Degradation of β-TCP implants in concurrent with replacement by newly formed bone^†^ | | |
| *25%-50%-75% degradation (mixed granules)* | 964.5 - 1173 - 1381 | 0.3 |
| *25%-50%-75% degradation (porous rod)* | 727.5 - 1015 - 1302 | 0.3 |

Notes: * The volume ratio of porous granules and dense granules is 12:5; ^†^The value of Young's modulus was estimated by the following formula: $Ec=Ea\times a\%+Eb\times b\%$, where “Ea” and “Eb” represent the Young's modulus of the two types of materials, “a%” and “b%” refer to the volume proportion of each material. “Ec” refers to the Young's modulus of the composite.
